# Clonally expanded alpha-chain T-cell receptor (TCR) transcripts are present in aneurysmal lesions of patients with Abdominal Aortic Aneurysm (AAA)

**DOI:** 10.1371/journal.pone.0218990

**Published:** 2019-07-16

**Authors:** Song Lu, John V. White, Raquel I. Judy, Lisa L. Merritt, Wan Lu Lin, Xiaoying Zhang, Charalambos Solomides, Ifeyinwa Nwaneshiudu, John Gaughan, Dimitri S. Monos, Emilia L. Oleszak, Chris D. Platsoucas

**Affiliations:** 1 Department of Microbiology and Immunology, Lewis Katz School of Medicine at Temple University, Philadelphia, PA, United States of America; 2 Department of Surgery, Advocate Lutheran General Hospital and University of Illinois School of Medicine, Park Ridge, IL, United States of America; 3 Department of Biological Sciences, Old Dominion University, Norfolk, VA, United States of America; 4 Department of Pathology and Laboratory Medicine, Lewis Katz School of Medicine at Temple University, Philadelphia, PA, United States of America; 5 Biostatistics Consulting Center, Lewis Katz School of Medicine at Temple University, Philadelphia, PA, United States of America; 6 Department of Pathology and Laboratory Medicine, The Children’s Hospital of Philadelphia and the Perelman School of Medicine, University of Pennsylvania, Philadelphia, PA, United States of America; 7 Department of Anatomy and Cell Biology, Lewis Katz School of Medicine at Temple University, Philadelphia, PA, United States of America; 8 Center for Molecular Medicine, Old Dominion University, Norfolk, VA, United States of America; Leiden University Medical Center, NETHERLANDS

## Abstract

Abdominal aortic aneurysm (AAA) is a life-threatening immunological disease responsible for 1 to 2% of all deaths in 65 year old or older individuals. Although mononuclear cell infiltrates have been demonstrated in AAA lesions and autoimmunity may be responsible for the initiation and account for the propagation of the disease, the information available about the pathogenesis of AAA is limited. To examine whether AAA lesions from patients with AAA contain clonally expanded α-chain TCR transcripts, we amplified by the non-palindromic adaptor-PCR (NPA-PCR)/Vα-specific PCR and/or the Vα-specific PCR these α-chain TCR transcripts. The amplified transcripts were cloned and sequenced. Substantial proportions of identical α-chain TCR transcripts were identified in AAA lesions of 4 of 5 patients, demonstrating that clonally expanded T cells are present in these AAA lesions. These results were statistically significant by the bimodal distribution. Three of 5 of these patients were typed by DNA-based HLA-typing and all three expressed DRB1 alleles containing the DRβGln70 amino acid residue that has been demonstrated to be associated with AAA. All three patients exhibited clonally expanded T cells in AAA lesions. Four of the 5 patients with AAA who exhibited clonal expansions of α-chain TCR transcripts, also exhibited clonal expansions of β-chain TCR transcripts in AAA lesions, as we have demonstrated previously (J Immunol 192:4897, 2014). αβ TCR-expressing T cells infiltrating AAA lesions contain T-cell clones which have undergone proliferation and clonal expansion *in vivo* in response to as yet unidentified specific antigens that may be self or nonself. These results provide additional evidence supporting the hypothesis that AAA is a specific antigen-driven T-cell autoimmune disease.

## Introduction

Abdominal aortic aneurysm (AAA) is a common immunological disease with a strong genetic component diagnosed in 3% of 60 years old or older [1–6]. AAA accounts for 1–2% of deaths of 65 years old or older men, and is the 13^th^ cause of death of men and women in the US of all ages [2]. AAA is characterized by dilations and enlargement of abdominal aorta with a diameter >3 cm or >50% of normal arteries [7]. Mortality of ruptured AAA is 85–90% [7,8].

Our understanding of the pathogenesis of AAA is limited. Environmental and genetic factors are involved and AAA is a complex multifactorial disease [3–5,9]. Strong evidence demonstrates that autoimmunity may be responsible for the pathogenesis of AAA and is summarized in Table 1 [3–5,9,10].

**Table 1:** Evidence demonstrating that autoimmunity may be responsible for the pathogenesis of AAA.

	References
T, B, and NK cells and monocyte/macrophages infiltrate AAA lesions.	[10–12]
Mononuclear infiltrating cells express early (CD69), intermediate (CD38, CD25) and late (CD45RO, HLA Class II) activation antigens revealing an ongoing immune response.	[10]
APCs are present in AAA lesions, often in physical contact with CD4+, CD8+ and B-lymphocytes.	[3, 13, 14]
Pro-inflammatory Th1 cytokines (IFN-γ, IL-2, and others) play an important role in the destruction of the aortic wall and the pathogenesis of AAA. Th2 cytokines are also present in AAA lesions.	[3, 9, 15–17]
IgG autoantibody from AAA lesions recognize proteins present in normal aortic tissue.	[18, 19]
AAA is associated with HLA class I (HLA-A2, HLA-B61) and class II (HLA-DRB1*02, -DRB1*04).	[20–22]
Several putative AAA self-antigens have been identified including elastin and elastin fragments microbial-associated glycoprotein-36, collagen types I and III, carbonic anhydrase and oxidized low-density lipoprotein.	[3, 18, 19, 23–30]
Several putative AAA nonself (microbial) antigens have also been identified, including *Chlamydia pneumonia*, *Treponema palladium* and cytomegalovirus. Molecular mimicry may be responsible for T-cell responses in AAA patients.	[31–36, 35, 37]
The frequency and the suppressor activity of CD4+CD25+FOXP3+ Tregs and the expression of FOXP3 transcript/protein are significantly lower in AAA vs normal donors.	[38–40]
Substantial proportions of identical β-chain T-cell receptor (TCR) transcripts are present in AAA lesions.	[10, 41]

Our findings of substantial proportions of identical β-chain TCR transcripts in AAA lesions demonstrate the presence of clonally expanded T cells [10, 41]. These results are statistically significant, can be explained only by proliferation and clonal expansion *in vivo* of T cell clones in response to specific, as yet unidentified antigen(s) [10, 41], and strongly suggest that AAA is a specific antigen driven T cell disease [10,41].

Two highly polymorphic polypeptides, the α- and the β- chain, are used by the TCR to recognize peptides in association with self-MHC, and are rearranged exclusively in T cells [42]. We have shown that clonally expanded β-chain TCR transcripts are present in AAA lesions [10,41], however, this β-chain TCR clonality does not necessarily imply clonality of the α-chain TCR. Experimental proof is needed. The α-chain TCR clonality and repertoire should also be studied in AAA lesions for several reasons (Table 2).

**Table 2:** Reasons to study α-chain TCR clonality and repertoire in AAA lesions.

	References
Both α- and β-chain TCR and in particular their CDR3s are critical in antigen recognition.	[43–46]
MHC class I & II restriction is mostly controlled by α-chain CDR1 and CDR3.	[42–49]
CD45RO^-^ T cell clones specific for a single antigenic epitope employ a unique TCR β-chain and may be paired on the average with 25 different TCR α-chains; CD45RO^+^ T-cell clones employ a TCR β-chain paired only with a single TCR α-chain	[50–52]
T cells expressing invariant α-chain TCR, but non-invariant β-chain TCR have been reported. Invariant natural killer T (NKT) cells utilize invariant α-chain TCR transcripts in both mice (Vα14Jα18) and humans (Vα24Jα18) and a limited number non-invariant β-chain TCR transcripts preferentially utilizing Vβ2 or Vβ7 (mice) and Vβ11 (humans).	[53, 54]
HLA Class II DRα of DR molecule is monomorphic, while DQα of DQ and DPα of DP molecules, respectively, are polymorphic. HLA class II are close counterparts of TCR in antigen recognition.	[55]
HLA class I are comprised of a polymorphic α-chain and an invariant β2-microglobulin, which does not involve directly in antigen recognition but supports structurally the α-chain.	[55]
Expression in appropriate T cells of clonally expanded α- and β-chain TCR in AAA lesions and development of an antigen presentation system may permit the identification of putative AAA antigen(s).	[56]

We report here that T cells infiltrating AAA lesions from patients with AAA contain clonally expanded α-chain TCR transcripts. Amplification of α-chain TCR transcripts from AAA lesions by the non-palindromic adaptor-PCR (NPA-PCR)/Vα-specific PCR and/or the Vα-specific PCR [41, 57–63] followed by cloning and sequencing demonstrated substantial proportions of identical α-chain TCR transcripts suggesting the presence of oligoclonal T cells. These results can be explained only by proliferation and clonal expansion of T-cell clones in vivo in response to specific, although not yet identified, self or non-self antigen(s), that they recognize.

## Methods

### Patients

The characteristics of the patients who provided AAA specimens for these studies are shown in Table 3. AAA size, race, age, gender, recent and past history of cardiovascular risk factors (high cholesterol, aortic valve replacement and current or ex-tobacco smoking) and associated diseases (chronic obstructive pulmonary disease, chronic renal disease, coronary artery disease, diabetes mellitus, and hypertension) are shown (Table 3). These patients were undergoing surgery for infrarenal AAAs repair. Adherent blood clots were stripped away from the aneurysm wall of these AAA specimens prior to use. Grossly normal infrarenal abdominal aortic specimens were obtained at autopsy from patients who died of nonvascular causes and employed as normal controls. Human peripheral blood mononuclear cells (PBMC) were employed as methodological control and were obtained from normal donors. The studies reported here were reviewed and approved by the IRB of Temple University Hospital and by the IRB of the Advocate Lutheran General Hospital. Written informed consent was obtained from the study participants. Procurement of additional peripheral blood from normal donors to prepare PBMC (methodological control) was approved by Old Dominion University.

**Table 3:** Characteristics of the patients with AAA*.

Patient	Gender	Race	Age	AAA size (cm)	HTN	COPD	TOB	CHOL	DM	Other
AAA00	M	C	71	4.8	Y	N	N	N	N	
AAA03	M	C	80	5.5	Y	N	N	Y	N	
AAA09	M	C	78	7.4	Y	Y	N	N	N	CAD/CRI
AAA10	M	C	78	7.9	N	N	N	Y	Y	CAD
AAA12	M	C	77	UN	UN	UN	UN	UN	UN	UN
	M = 5	C = 5	Avg. = xx n = 5	Avg. = xx n = 4 UN = 1	Yes = 3 No = 1 UN = 1	Yes = 1 No = 3 UN = 1	Yes = 0 No = 4 UN = 1	Yes = 2 No = 2 UN = 1	Yes = 1 No = 3 UN = 1	

M: Male; UN: Unknown; C: Caucasian; Y: Yes; N: No.

HTN: Hypertension; COPD: Chronic obstructive Pulmonary Disease; TOB: Current or ex- tobacco smoker; CHOL: High cholesterol; DM: Diabetes mellitus; CAD: Coronary artery disease; CRI: Chronic renal disease; AOVR: Aortic valve replacement.

* Reproduced in part, with the permission of The American Association of Immunologists, Inc., from Lu et al, J. Immunol., 192: 4897–4912, 2014 (reference [41]). Copyright 2014. The American Association of Immunologists, Inc.

### Immunohistochemistry

AAA specimens were divided into two fractions. One was used for RNA preparation either immediately (fresh) or it was snap frozen in liquid nitrogen and used at a later time. The other fraction was embedded in optimum cutting technology (OCT) formulation and it was snap frozen in liquid nitrogen and stored, until used, at -70°C for immunohistochemistry. Immunostaining was performed as described [64–66], by the avidin-biotin complex (ABC)—immunoperoxidase method (Vector Labs, Burlingame, CA), using: (i) an anti-CD3 monoclonal antibody (mab), clone NCL-CD3-PS1 (Novocastra, Newcastle upon Tyne, U.K.); (ii) an anti-CD4 mab clone 4B12 (Dako Glustrop, Denmark); (iii) an anti-CD8 mab clone C8/144B (Dako).

### Isolation of PBMC from normal donors

PBMC were isolated from venous peripheral blood by a Ficoll-Hypaque density cushion [67].

### DNA-based HLA-typing for HLA-DRB1, -DQA1 and -DQB1

DNA was prepared from AAA specimens for HLA-typing of DRB1, DQA1 and DQB1 loci as previously described [68]. Typing at HLA-DRB1 (exon 2) and DQB1 (exons 2 and 3) using AlleleSEQR typing reagents (Abbott Molecular, Des Plaines, IL). Typing at HLA-DQA1 was carried out using sequence specific primers (SSP) typing (Invitrogen SSP-Unitrays, Carlsbad, CA; Qiagen Olerup-SSP, Valencia, CA). Any remaining ambiguities of HLA-typing were resolved using sequence specific primers (SSP) typing (Invitrogen SSP-Unitrays, Carlsbad, CA; Qiagen Olerup-SSP, Valencia, CA). DNA sequencing was carried out using an ABI 3130 sequencer (Applied Biosystems, Carlsbad, CA). Results were analyzed by Assign-SBT v3.5 Software (Conexio Genomics, Fremantle, Australia).

### RNA isolation

Total RNA was prepared from fresh (cryopreserved) AAA lesions containing tissue from these patients using a guanidinium thiocyanate solution, as recommended by the manufacturer (Stratagene, La Jolla, CA), and then treated with DNase from the Atlas pure total RNA labeling system (Clontech Laboratories, Inc., Mountain View, CA) to eliminate potential genomic DNA contamination.

### Synthesis of cDNA

cDNA was synthesized from oligo-(dT)15- *Not*I (Promega) primed total RNA. We employed a SuperScript II (GibcoBRL) cDNA synthesis kit, according to the manufacturer’s specifications [41,57–63]. Double-stranded cDNA was blunt-ended for efficient adaptor ligation by using T4 DNA polymerase.

### Amplification by the nonpalindromic adaptor PCR (NPA-PCR)/Vα-specific PCR

#### (i) Adaptor ligation and NotI digestion

Double-stranded blunt-ended cDNA was ligated at the 5’ and 3’ blunt ends with an equivalent molar concentration of a nonpalindromic adaptor (NPA) comprised of two complementary oligonucleotides pre-annealed to each other: 5’-AATTCGAACCCCTTCGAGAATGCG-3’ and 3’-GCTTGGGGAAGCTCTTACGC-p-5’ (S1 Table). cDNA and NPA were ligated for 14 hrs at 16°C with T4 DNA ligase (Gibco-BRL). The ligated adaptor was removed from the 3’ end of the double-stranded cDNA by *Not*I restriction endonuclease digestion (2 hrs at 37°C), while it was retained at the 5’ end [41,57–63]. Digested cDNA was purified using a G-50 column as recommended (5’-3’, Boulder, Co).

#### (ii) First cycle amplification by NPA-PCR

This was carried out as described [41,57–63], with minor modifications. The nonpalindromic adaptor 5’-AATTCGAACCCCTTCGAGATGCT-3’ oligonucleotide was employed as the 5’ amplification primer. A hCα3 oligonucleotide located in the Cα region, 3’ of Jα, was employed as a 3’ amplification primer (S1 Table). cDNA (20 μl), purified by a G-50 column, was amplified using NPA-PCR, in 100μl, containing the cDNA, the amplification primers, 5 units of native pfu DNA polymerase and 1mM dNTPs in 1xbuffer [41,57–63]. PCR was performed, as follows: 5 min at 95°C for cDNA denaturation, amplification by 30 cycles including 1 min at 94°C (denaturation), 1 min at 45°C (annealing), 2 min at 72°C (elongation) and a final extension of 7 min at 72°C. The amplified transcripts were purified by a G-50 column, as recommended by the manufacturer.

#### (iii) Second cycle of amplification by individual Vα-specific PCR

Thirty two different Vα-specific PCRs were performed. NPA-PCR amplified α-chain TCR cDNA (4 μl) was used as a template in a reaction volume of 50 μl, which contained the cDNA, the amplification primers, 2.5 units of native pfu DNA polymerase and 1mM dNTPs in 1xbuffer [41,57–63]. Single oligonucleotides, each specific for one of 32 Vα families (S1 Table), were used in 32 separate amplifications, as 5’ end amplification primer. A Cα 3’amplification primer designated as hCα2 was used and it is located 5’ to the hCα3 primer employed for the first NPA-PCR amplification (nested design), 3’ of Jα (S1 Table). This nested design virtually eliminates possible PCR amplification of members of the Ig supergene family that may share homology with the α-chain TCR because it will be unlikely that these members will have substantial homology with both hCα3 and hCα2 sites. The reaction mixture was denatured at 95°C for 5 min, amplified by 30 PCR cycles at 94°C for 1 min, 55°C for 1 min, and 72°C for 2 min followed by a 7 min final extension step at 72°C.

### Single Vα-specific PCR amplification

This was performed [41,57–63] to evaluate, in more detail, single Vα families or subfamilies or to confirm clonal expansions identified by NPA-PCR/Vα-specific PCR. Template cDNA was synthesized from total RNA isolated from the same AAA specimen employed for NPA-PCR/Vα-specific PCR, or from total RNA prepared in separate experiments from the same AAA specimen. Selection of the Vα families to be amplified by Vα-specific PCR was made on the basis of the NPA-PCR/Vα-specific PCR findings. Vα5 and Vα24 (patient AAA03), Vα6, Vα9 and Vα12 (patient AAA09), Vα2 and Vα8 (patient AAA00) and Vα6 (patient AAA10) families were amplified by Vα-specific PCR. Vα family specific oligonucleotides (S1 Table), were used as 5’ end amplification primers. The hCα2 oligonucleotide was used as 3’amplification primer. Vα-specific PCR (35 cycles), included denaturation (94°C, 1 min), annealing (55°C, 1 min), elongation (72°C, 2 min), and final extension (72°C, 7 min).

### Cloning of the PCR products

Eight μl from each one of the 32 NPA-PCR/Vα-specific PCR amplified products were mixed together and incubated with Taq polymerase for 10 min at 72°C to add an adenine at the 3’ end and the mixture was selected by agarose gel electrophoresis in respect to size and purified using a Geneclean Kit (Bio101, Vista, CA), following the instructions of the manufacturer. Mixing these PCR amplification products from each of the 32 Vα families before cloning and sequencing, reduces substantially the work needed for these experiments [41,57–59]. Purified NPA-PCR/Vα-specific PCR or Vα-specific PCR products were cloned into the TOPO-TA vector (Invitrogen, Carlsbad, CA), were transformed into Top10 One Shot Chemically competent cells (Invitrogen) as described by the manufacturer and were submitted to blue-white screening. Top10 One Shot Chemically competent *E*.*coli* cells were incubated for 30–45 min with the vector on ice and were subjected to heat shock for 30–45 sec at 42°C. The competent cells were incubated on ice for 2 min and 250 μl of SOC medium were added and incubated at 37°C for 1 hr. The competent cells were plated in X-gal containing agar plates [41,57–63]. White colonies were collected using the Perfectprep Plasmid Mini Kit (Eppendorf, Westbury, NY) following the specifications of the manufacturer. Large numbers of white colonies were obtained.

The TCR repertoire is very large [47–49,69,70] and for this reason the probability is very small to find by chance in an independent sample of T lymphocytes two identical copies of a single α- or β-chain TCR transcript. The only mechanism that can explain the presence of multiple identical copies of a single α- or β-chain TCR transcript in an independent population of T lymphocytes is specific antigen-driven proliferation and clonal expansion (reviewed in [56]). However, during transformation of DH5α-*E*. *coli*-competent cells the mixture of *E*. *coli* cells and plasmid was heat shocked by treatment for 30 sec at 42°C followed by growth in SOC medium at 37°C for 1 hr before the colonies were plated. In ideal growth conditions (log phase growth) *E*. *coli* cells divide every 20 min and this could result in two divisions in one hour [71]. Because of the heat shock treatment, the *E*. *coli* cells may not enter right away to the logarithmic growth phase. However, it is possible, although unlikely, that few *E*. *coli*-transformed cells may double before they are plated. Therefore, the appearance in two different colonies of an identical TCR transcript, designated as a doublet, may indicate a clonal expansion or may be the result of a single *E*. *coli* cell transformed and divided (doubled) before it was plated. We are addressing this issue in the statistical method that we are employing (see below; second alternative hypothesis), although doubling of a transfected *E*. *coli* cell before plating is rather infrequent. As a methodological control, to make certain that all PCR, cloning and sequencing methods were performing correctly, we amplified by NPA-PCR/Vα-specific PCR or NPA-PCR, cloned, and sequenced 125 α-chain TCR transcripts from PBMC of 3 normal donors. An additional 45 α-chain TCR transcripts from PBMC of 2 normal donors were reported previously (refs. [59] and [60]) (see below).

### Sequencing

Plasmids were sequenced by the dideoxy chain termination method using 6% polyacrylamide DNA sequencing gels and an ABI373A DNA Sequencer (Applied Biosystems, Foster City, CA). Comparable numbers of α-chain TCR clones were obtained after NPA-PCR/Vα-specific PCR amplification and cloning and as well as after Vα-specific PCR and cloning.

### Computer analysis. Sequence comparison

Sequences of α-chain TCR transcripts were identified in AAA lesions or in PBMC from normal controls encoding for V, D, J and C regions by comparing them to those in the NCBI databases by the standard nucleotide-nucleotide BLAST sequence alignment program [41,57–63]. The N region nucleotide sequence of TCR transcripts was identified as the nucleotide sequence contained between the last identifiable Vα nucleotide and the first identifiable Jα nucleotide. Deduced CDR3 region amino acid sequences were compared to those in the NCBI databases using BLAST gapped BLAST and PSI BLAST protein database programs.

### Statistical analysis

The binomial distribution was employed [41,58,59,62] to determine the probability, p, that the number (x) of the multiple identical α-chain TCR transcripts identified among those sequenced (x/n; n is the total number of α-chain TCR transcripts sequenced), was statistically significant versus (i) a first alternative hypothesis that each α-chain TCR transcript is expressed only once and all α-chain TCR transcripts sequenced when compared to each other are unique (1/n), or (ii) a second alternative hypothesis that only a single α-chain TCR transcript is expressed twice and all the remaining α-chain TCR transcripts identified are expressed only once (2/n) [41]. The first alternative and the second alternative hypotheses were initially developed [41] for the analysis of β-chain TCR, and their use is extended here to the analysis of α-chain TCR.

## Results

### Multiple identical copies of α-chain TCR transcripts are present in AAA lesions

Immunohistochemical staining of AAA tissues with the anti-CD3 mab revealed the presence of substantial CD3+ T-cell infiltrates in these AAA lesions which were predominant in the adventitia and media [10, 41]. Staining with the appropriate mabs revealed that these infiltrates contained both CD4+ and CD8+ T lymphocytes [41]. These results have been shown previously [10,41] and they are in agreement with the reports of others [11–14]. Rare, if any, mononuclear cell infiltrates are found in grossly normal autopsy infrarenal abdominal aortic tissue which was obtained from patients who died of nonvascular causes [41].

Multiple identical copies of α-chain TCR transcripts were found in AAA lesions from patients with AAA by PCR amplification, cloning and sequencing. Comparison of these sequences to those of the GenBank database using BLAST revealed that they were novel (not reported in the GenBank) and typical of productively rearranged human α-chain TCR transcripts. Statistically significant, by the binomial distribution, α-chain TCR clonal expansions were identified in AAA lesions in 4 of 5 patients with AAA.

Sequence analysis of α-chain TCR transcripts from AAA lesions from patient AAA03 after NPA-PCR/Vα-specific PCR amplification followed by cloning and sequencing revealed several clonal expansions (Table 4): (i) Clone aaa03npa04 accounted for 5 of 57 identical transcripts (9%) (Vα5.1Jα3) (CDR3: LE). These results are statistically significant by the bimodal distribution. Probability (p) of appearance of 5 of 57 identical α-chain TCR transcripts by chance against the alternative hypothesis that each α-chain TCR transcript is expressed only once 1/n = 1/57, was p = 0.003. The p of the appearance of 5 of 57 identical α-chain TCR transcripts against the alternative hypothesis that only a single α-chain TCR is expressed twice, 2/n = 2/57, and all remaining α-chain TCR transcripts sequenced were expressed only once, was p = 0.034; (ii) Clone aaa03npa21 accounted for 5 of 57 identical transcripts (9%)(p = 0.003 vs. 1/57 and p = 0.034 vs. 2/57)(Vα24.1Jα47) (CDR3: AGR); (iii) clone aaa03npa09 accounted for 4 of 57 identical transcripts (7%)(p = 0.015 vs. 1/57 and p = 0.089 vs. 2/57)(Vα14.1Jα47)(CDR3:IS); (iv) clone aaa03npa33 accounted for 4 of 57 identical transcripts (7%)(p = 0.015 vs. 1/57 and p = 0.089 vs. 2/57) (Vα6.1Jα54) (CDR3: EGE). (v) Clones aaa03npa16 and aaa03npa02 accounted each for 3 of 57 identical transcripts (p = 0.06); six clones were expressed in duplicate and the remaining 21 clones were unique when compared to each other. Eighteen of 57 of those clones are shown in the supporting information section (S2 Table).

**Table 4:** α-chain TCR transcripts (CDR3 region) expressed in aneurysmal wall of patients with AAA.

Clone	Vα	N	Jα	Transcript Frequency in Specimen	p value	
**PATIENT AAA03**						
**α-chain TCR transcripts amplified by NPA- PCR/Vα-specific PCR**					**vs.** **1/57**	**vs.** **2/57**
aaa03npa04	**C A**	**L E**	**G Y S S**	Vα5.1Jα3	*p* = 0.003	*p* = 0.034
	tgtgct	ctaga	ggggtacagcagt	5/57(9%)		
aaa03npa21	**C V V S**	**A G R**	**E Y G N K L**	Vα24.1Jα47	*p* = 0.003	*p* = 0.034
	tgtgtggtgagc	gcgggtag	ggaatatggaaacaaactg	5/57(9%)		
aaa03npa09	**C A Y**	**I S**	**G N K L**	Vα14.1Jα47	*p* = 0.015	*p* = 0.089
	tgtgcttat	atctc	tggaaacaaactg	4/57(7%)		
aaa03npa33	**C A M R**	**E G E**	**G G S N Y K L**	Vα6.1Jα54	*p* = 0.015	*p* = 0.089
	tgtgcaatgaga	gagggcgag	ggaggtagcaactataaactg	4/57(7%)		
aaa03npa16	**C A V**	**E**	**E T S G S R L**	Vα13.1Jα58	*p* = 0.06	*p* = 0.183
	tgtgctgtg	g	aagaaaccagtggctctaggttg	3/57(5%)		
aaa03npa02	**C A G**	**Q K**	**G G T S Y G K L**	Vα25.1Jα53	*p* = 0.06	*p* = 0.183
	tgtgctggg	cagaag	ggtggtactagctatggaaagctg	3/57(5%)		
aaa03npa19	**C A V E**	**T**	**G F Q K L**	Vα11.1Jα8	ns	ns
	tgtgctgtggag	acc	ggctttcagaaactt	2/57(4%)		
aaa03npa12	**C A V**	**V R**	**N N N D R L**	Vα13.1Jα31	ns	ns
	tgtgctgtc	gttc	ggaataacaatgacagactc	2/57(4%)		
aaa03npa10	**C A V**	**V T S V**	**G S G N T G K L**	Vα13.1Jα37	ns	ns
	tgtgctgtg	gtcactagcgt	tggctctggcaacacaggcaaacta	2/57(4%)		
aaa03npa26	**C A V**	**A K D**	**G G S Q G N L**	Vα13.1Jα42	ns	ns
	tgtgctgtg	gcaaagg	atggaggaagccaaggaaatctc	2/57(4%)		
aaa03npa11	**C A V**	**E G**	**S S G D K L**	Vα13.1Jα46	ns	ns
	tgtgctgtg	gaggg	aagcagcggagacaagctg	2/57(4%)		
aaa03npa20	**S A V Y**	**P S P V A**	**G G G N K L**	Vα21.1Jα10	ns	ns
	tctgcagtgtac	ccatctcccgtcg	cgggaggaggaaacaaactc	2/57(4%)		
aaa03npa27	**C A T**	**D S F V Y S N**	**A S K I**	Vα3.1Jα3	ns	ns
	tgtgctacg	gactctttcgtttacagcaa	tgcttccaagata	1/57(2%)		
aaa03npa61	**C A T**	**A R**	**M D S S Y K L**	Vα3.1Jα12	ns	ns
	tgtgctacg	gccc	ggatggatagcagctataaattg	1/57(2%)		
aaa03npa36	**C A T**	**D A**	**N D Y K L**	Vα3.1Jα20	ns	ns
	tgtgctacg	gacgca	aacgactacaagctc	1/57(2%)		
Remaining 18 of 57 sequences are unique when compared to each other and are shown in the supporting information section, S2 Table.						
**α-chain TCR transcripts amplified by single Vα5-specific PCR**					vs. 1/20	vs. 2/20
aaa03npa04	**C A**	**L E**	**G Y S S A S K I**	Vα5.1Jα3	*p*<0.0001	*p*<0.0001
	tgtgct	ctaga	ggggtacagcagtgcttccaagata	20/20(100%)		
**α-chain TCR transcripts amplified by single Vα24-specific PCR**					vs. 1/22	vs. 2/22
aaa03npa21	**C V V S**	**A G R**	**E Y G N K L**	Vα24.1Jα47	*p*<0.0001	*p*<0.0001
	tgtgtggtgagc	gcgggtag	ggaatatggaaacaaactg	13/22(59%)		
aaa03va2401	**C V V S**		**S G G S Y I**	Vα24.1Jα6	*p* = 0.0003	*p* = 0.0092
	tgtgtggtgagc		tcaggaggaagctacata	6/22(27%)		
aaa03va2408	**C V**	**A A**	**T G G F K T**	Vα24.1Jα9	*p* = 0.06	*p* = 0.189
	tgtgtg	gcggc	tactggaggcttcaaaact	3/22(14%)		
**PATIENT AAA09**						
**α-chain TCR transcripts amplified by NPA-PCR/Vα-specific PCR**					vs. 1/36	vs. 2/36
aaa09npa08	**C A L S**	**E P F**	**Q T G A N N L**	Vα12.1Jα36	*p* = 0.06	*p* = 0.187
	tgtgctctgagt	gagccttt	tcaaactggggcaaacaacctc	3/36(8%)		
aaa09npa16	**C A L**	**S R**	**G S Q G N L**	Vα9.1Jα42	*p* = 0.06	*p* = 0.187
	tgtgctctg	tcta	gaggaagccaaggaaatctc	3/36(8%)		
aaa09npa01	**C A M R**	**E G D**	**Q A G T A L**	Vα6.1Jα15	ns	ns
	tgtgcaatgaga	gagggag	accaggcaggaactgctctg	2/36(8%)		
aaa09npa37	**C A M R**	**E I R**	**T S Y D K V**	Vα6.1Jα50	ns	ns
	tgtgcaatgaga	gagattcgg	acctcctacgacaaggtg	2/36(6%)		
aaa09npa22	**C A L**	**R G L**	**N Q A G T A L**	Vα9.1Jα15	ns	ns
	tgtgctcta	aggggtctc	aaccaggcaggaactgctctg	2/36(6%)		
aaa09npa39	**C A L**	**A P**	**S G N T G K L**	Vα5.1Jα37	ns	ns
	tgtgctcta	gcccc	ctctggcaacacaggcaaacta	1/36(3%)		
aaa09npa26	**C A M R**	**S**	**N T G F Q K L**	Vα6.1Jα8	ns	ns
	tgtgcaatgaga	tc	gaacacaggctttcagaaactt	1/36(3%)		
aaa09npa09	**C A M R**	**E G M A**	**G F Q K L**	Vα6.1Jα8	ns	ns
	tgtgcaatgaga	gagggcatgg	caggctttcagaaactt	1/36(3%)		
Remaining 21 of 36 sequences are unique when compared to each other and are shown in the supporting information section, S3 Table.						
**α-chain TCR transcripts amplified by single Vα12-specific PCR**					vs. 1/20	vs. 2/20
aaa09npa08	**C A L S**	**E P F**	**Q T G A N N L**	Vα12.1Jα36	*p*<0.0001	*p*<0.0001
	tgtgctctgagt	gagccttt	tcaaactggggcaaacaacctc	11/20(55%)		
aaa09va1201	**C A L**	**R G T**	**N D Y K L**	Vα12.1Jα20	ns	ns
	tgtgctctg	aggggga	ctaacgactacaagctc	2/20(10%)		
aaa09va1207	**C A L S**	**A G A S**	**S S A S K I**	Vα12.1Jα3	ns	ns
	tgtgctctgagt	gcaggcgcgtc	cagcagtgcttccaagata	1/20(5%)		
aaa09va1219	**C A L S**	**R G**	**G N N R L**	Vα12.1Jα7	ns	ns
	tgtgctctgagt	cgtgg	tgggaacaacagactc	1/20(5%)		
Remaining 5 of 20 sequences are unique when compared to each other and are shown in the supporting information section, S3 Table.						
**α-chain TCR transcripts amplified by single Vα6-specific PCR**					vs. 1/21	vs. 2/21
aaa09npa37	**C A M R**	**E I R**	**T S Y D K V**	Vα6.1Jα50	*p* = 0.06	ns
	tgtgcaatgaga	gagattcgg	acctcctacgacaaggtg	3/21(14%)		
aaa09va0609	**C A M R**	**E G L**	**Y G G A T N K L**	Vα6.1Jα32	ns	ns
	tgtgcaatgaga	gagggcctc	tatggaggtgctacaaacaagctc	2/21(10%)		
aaa09va0602	**C A M R**	**G**	**G F G N V L**	Vα6.1Jα35	ns	ns
	tgtgcaatgaga	ggg	ggctttgggaatgtgctg	2/21(10%)		
aaa09va0612	**C A M R**	**E G P**	**G Y S S A S K I**	Vα6.1Jα3	ns	ns
	tgtgcaatgaga	gagggccct	gggtacagcagtgcttccaagata	1/21(5%)		
aaa09va0608	**C A M**	**S P N R**	**G G Y N K L**	Vα6.1Jα4	ns	ns
	tgtgcaatg	agccctaacagg	ggtggctacaataagctg	1/21(5%)		
aaa09va0607	**C A M R**	**V N Y R A**	**R R A L**	Vα6.1Jα5	ns	ns
	tgtgcaatgaga	gtaaactatcggg	cgggcaggagagcactt	1/21(5%)		
Remaining 11 of 21 sequences are unique when compared to each other and are shown in the supporting information section, S3 Table.						
**α-chain TCR transcripts amplified by single Vα9-specific PCR**					vs. 1/19	vs. 2/19
aaa09npa16	**C A L**	**S R**	**G S Q G N L**	Vα9.1Jα42	*p* = 0.06	ns
	tgtgctctg	tcta	gaggaagccaaggaaatctc	3/19(16%)		
aaa09npa12	**C A L**	**R F R A**	**Y S S A S K I**	Vα9.1Jα3	ns	ns
	tgtgctctg	cgattccgtgcc	tacagcagtgcttccaagata	2/19(11%)		
aaa09va0906	**C A L**	**S**	**G S G N T G K L**	Vα9.1Jα37	ns	ns
	tgtgctcta	a	gtggctctggcaacacaggcaaacta	2/19(11%)		
aaa09npa24	**C A L S**	**R S G T**	**R L**	Vα9.1Jα58	ns	ns
	tgtgctctaagt	cggtccggga	ctaggttg	2/19(11%)		
aaa09va0902	**C A L S**	**G D**	**N F N K F**	Vα9.1Jα21	ns	ns
	tgtgctctaagt	gggg	acaacttcaacaaattt	1/19(5%)		
aaa09npa42	**C A**	**Q S P**	**G Y N K L**	Vα9.1Jα4	ns	ns
	tgtgct	cagtccc	ctggtggctacaataagctg	1/19(5%)		
aaa09va0905	**S A**	**S E G H**	**G G S Q G N L**	Vα9.1Jα42	ns	ns
	tcagcc	tcagaaggtc	atggaggaagccaaggaaatctc	1/19(5%)		
Remaining 7 of 19 sequences are unique when compared to each other and are shown in the supporting information section, S3 Table.						
**PATIENT AAA00**						
**α-chain TCR transcripts following Vα2-specific PCR**					vs. 1/15	vs. 2/15
aaa00va0201	**C V V**	**T G**	**T G G F K T**	Vα2.3Jα9	*p*<0.0001	*p*<0.0001
	tgtgtggtg	acggga	actggaggcttcaaaact	15/15 (100%)		
**α-chain TCR transcripts following Vα8-specific PCR**					vs. 1/15	vs. 2/15
aaa00va0801	**C A E**	**E**	**G G S N Y K L**	Vα8.2Jα54	*p*<0.0001	*p*<0.0001
	tgtgcagag	gag	ggaggtagcaactataaactg	15/15 (100%)		
**PATIENT AAA10**						
**α-chain TCR transcripts amplified by NPA-PCR/Vα-specific PCR**					vs. 1/30	vs. 2/30
aaa10npa03	**C A M**	**T P P G**	**G G T S Y G K L**	Vα6.1Jα53	*p* = 0.06	ns
	tgtgcaatg	acacctcccggg	ggtggtactagctatggaaagctg	3/30(10%)		
aaa10npa04	**C A M**	**R E T**	**N T D K L**	Vα6.1Jα34	ns	ns
	tgtgcaatg	agagaaacg	aacaccgacaagctc	2/30(7%)		
aaa10npa07	**C A**	**P F G G**	**S N S G Y A L**	Vα13.1Jα41	ns	ns
	tgtgct	cccttcggcggg	tcaaattccgggtatgcactc	2/30(7%)		
aaa10npa022	**C G A**	**D Y P**	**S G T Y K Y**	Vα26.1Jα40	ns	ns
	tgtggagca	gactatc	cctcaggaacctacaaatac	2/30(7%)		
aaa10npa09	**C A T**	**D Y**	**S G S A R Q L**	Vα3.1Jα22	ns	ns
	tgtgctacg	gactac	tctggttctgcaaggcaactg	1/30(3%)		
aaa10npa44	**C A T**	**P K**	**S G T Y K Y**	Vα3.1Jα40	ns	ns
	tgtgctacc	cctaag	tcaggaacctacaaatac	1/30(3%)		
aaa10npa43	**C A T**	**D A R H**	**S N S G Y A L**	Vα3.1Jα41	ns	ns
	tgtgctacg	gacgcccgcc	actcaaattccgggtatgcactc	1/30(3%)		
Remaining 18 of 30 sequences are unique when compared to each other and are shown in the supporting information section, S4 Table.						
**α-chain TCR transcripts following Vα6-specific PCR**					vs. 1/21	vs. 2/21
aaa10npa03	**C A M**	**T P P G**	**G G T S Y G K L**	Vα6.1Jα53	*p* = 0.01	*p* = 0.09
	tgtgcaatg	acacctcccggg	ggtggtactagctatggaaagctg	4/21(19%)		
aaa10npa29	**C A M R**	**E A Y S**	**G N Q F Y**	Vα6.1Jα49	*p* = 0.01	*p* = 0.09
	tgtgcaatgaga	gaggcgtact	ccggtaaccagttctat	4/21(19%)		
aaa10va0601	**C A M R**	**E V D**	**T G G F K T**	Vα6.1Jα9	ns	ns
	tgtgcaatgaga	gaggtcg	atactggaggcttcaaaact	2/21(10%)		
aaa10va0610	**C A M R**	**E T**	**N T D K L**	Vα6.1Jα34	ns	ns
	tgtgcaatgaga	gaaacg	aacaccgacaagctc	2/21(10%)		
aaa10va0614	**C A M R**	**P R**	**S G Y S T L**	Vα6.1Jα11	ns	ns
	tgtgcaatgaga	ccgag	gaattcaggatacagcaccctc	2/21(10%)		
aaa10va0620	**C A M**	**S P**	**M D S S Y K L**	Vα6.1Jα12	ns	ns
	tgtgcaatg	agtcc	gatggatagcagctataaattg	1/21(5%)		
aaa10va0618	**C A M R**	**E A L**	**M D S S Y K L**	Vα6.1Jα12	ns	ns
	tgtgcaatgaga	gaggccctt	atggatagcagctataaattg	1/21(5%)		
aaa10va0602	**C A M R**	**D**	**G Q K L**	Vα6.1Jα15	ns	ns
	tgtgcaatgaga	ga	tggccagaagctg	1/21(5%)		
Remaining 4 of 21 sequences are unique when compared to each other and are shown in the supporting information section, S4 Table.						
**PATIENT AAA12**						
**α-chain TCR transcripts amplified by NPA-PCR/Vα-specific PCR**					vs. 1/31	vs. 2/31
aaa12npa26	**C A V**	**S A K**	**N N N A R L**	Vα13.1Jα31	ns	ns
	tgtgctgtg	tctgcgaa	gaataacaatgccagactc	2/31(6%)		
aaa12npa02	**C A**	**P E G**	**G G G A D G L**	Vα13.1Jα45	ns	ns
	tgtgcc	ccggagggg	ggaggaggtgctgacggactc	2/31(6%)		
aaa12npa16	**C G A**	**H S N**	**S G G G A D G L**	Vα26.1Jα45	ns	ns
	tgtggagcc	cactcaa	attcaggaggaggtgctgacggactc	2/31(6%)		
aaa12npa07	**C A L**	**R**	**N Q T G T A L**	Vα5.1Jα15	ns	ns
	tgtgctctc	cgt	aaccagacaggaactgctctg	1/31(3%)		
aaa12npa04	**C A L**	**E G D**	**N A G N M L**	Vα5.1Jα39	ns	ns
	tgtgctcta	gaagggg	ataatgcaggcaacatgctc	1/31(3%)		
aaa12npa28	**C A M**	**T S K T I**	**I F G Q**	Vα6.1Jα37	ns	ns
	tgtgcaatg	acctctaaaacaa	taatctttgggcaa	1/31(3%)		
Remaining 22 of 31 sequences are unique when compared to each other and are shown in the supporting information section, S5 Table.						

The Vα5.1Jα3 clonal expansion (clone aaa03npa04)(CDR3:LE), identified by NPA-PCR/Vα-specific PCR, was confirmed by Vα5-specific PCR followed by cloning and sequencing (Table 4). Twenty of 20 (100%) (p<0.0001) Vα5.1Jα3 transcripts were identical to the clonally-expanded aaa03npa04 clone which was initially identified by NPA-PCR/Vα-specific PCR (Table 4). The Vα24.1Jα47 clonal expansion (clone aaa03npa21) (CDR3: AGR) was confirmed by Vα24-specific PCR followed by cloning and sequencing. Thirteen of 22 (59%) (p<0.0001) Vα24.1 transcripts were identical to the clonally expanded aaa03npa21 clone, initially identified by NPA-PCR/Vα-specific PCR (Table 4). Another Vα24 clonal expansion was identified by Vα24-specific PCR followed by cloning and sequencing (clone aaa03Va2401) and accounted for 6 of 22 (27%) (p = 0.0003) identical transcripts (Vα24.1Jα6). Clone aaa03Va2408 (Vα24.1Jα9) was expressed in triplicate (Table 4).

Sequence analysis of α-chain TCR transcripts from AAA tissue from patient AAA09 after NPA-PCR/Vα-specific PCR followed by cloning and sequencing revealed (Table 4) the presence of: (i) clone (aaa09npa08) that accounted for 3 of 36 identical transcripts (8%)(Vα12.1Jα36)(CDR3: EPF) (p = 0.06); (ii) clone (aaa09npa16) that accounted for 3 of 36 identical transcripts (8%) (Vα9.1Jα42) (CDR3:SR) (p = 0.06). Three α-chain TCR clones were expressed in duplicate. Remaining 24 of 36 α-chain TCR clones were unique when compared to each other and are shown in Table 4 (3 clones)] and in the supporting information section (S3 Table)(21clones).

The Vα12.1Jα36 clonal expansion identified by NPA-PCR/Vα-specific PCR, was confirmed by Vα12-specific PCR followed by cloning and sequencing (Table 4). Eleven of 20 (55%) (p<0.0001) Vα12.1Jα36 transcripts were identical to the clonally-expanded aaa03npa08 clone identified by NPA-PCR/Vα-specific PCR (Table 4). One Vα12.1 clone was present in duplicate. Remaining 7 T-cell clones were unique when compared to each other and are shown in Table 4 and in S3 Table. Clone (aaa09npa23) identified (single copy) by NPA-PCR/Vα.12.1-specific PCR (S3 Table) was also found (single copy) after Vα12.1-specific PCR (S3 Table).

Clone aaa09npa37, Vα6.1Jα50 (CDR3: EIR) was expressed in triplicate and accounted for 3 of 21 identical transcripts (14%) (p = 0.06) as determined by Vα6-specific PCR amplification, cloning and sequencing (Table 4). The same clone Vα6.1Jα50 was found to be expressed in duplicate (2 of 36; 6%; p = 0.19) by NPA-PCR/Vα-specific PCR (Table 4). Remaining 14 Vα6.1 α-chain clones identified by Vα6-specific PCR, were unique when compared to each other and are shown in Table 4 (three) and S3 Table (eleven). However, three clones aaa09npa15, aaa09npa35 and aaa09npa45 were identified by both NPA-PCR/Vα-specific PCR and Vα6-specific PCR followed by cloning and sequencing and were present in single copies (Table 4 and S3 Table).

Several clones identified by NPA-PCR/Vα-specific PCR followed by cloning and sequencing were also identified by Vα9.1-specific PCR (Table 4). Clone aaa09npa16, Vα9.1Jα42 (CDR3:SR) accounted for 3 of 36 identical α-chain transcripts (8%) (p = 0.06) and was identified by NPA-PCR/Vα9.1-specific PCR. The same clone Vα9.1Jα42, CDR3:SR, was identified by Vα9.1-specific PCR and accounted for 3 of 19 identical α-chain TCR transcripts (16%) (p = 0.06). Clones aaa09npa16, aaa09npa24, aaa09npa42, and aaa09npa34 were identified by both NPA-PCR/Vα-specific PCR and Vα9.1-specific PCR amplification, 3 out of 4 were present in single copies, and one in duplicate (Table 4 and S3 Table). Seven of 19 clones were unique when compared to each other and are shown in S3 Table.

Vα2-specific PCR amplification of TCR transcripts from AAA lesions from patient AAA00 followed by cloning and sequencing demonstrated 15 of 15 identical Vα2-chain TCR transcripts (100%) (p<0.0001)(clone aaa00va0201)(Vα2.3Ja9)(Table 4). Vα8-specific PCR of TCR transcripts from AAA lesions from patient AAA00, followed by cloning and sequencing, revealed 15 of 15 identical Vα8-chain TCR transcripts (100%)(p<0.0001)(clone aaa00va0801)(Vα8.2Jα54) (Table 4).

Sequence analysis of α-chain TCR transcripts from AAA lesions from patient AAA10 after NPA-PCR/Vα-specific PCR amplification and cloning, showed 3 of 30 (10%) identical α-chain TCR transcripts (clone aaa10npa03)(p = 0.06)(Vα6.1Jα53)(CDR3:TPPG)(Table 4). Clones, aaa10npa04 (Vα6.1Jα34), aaa10npa07 (Vα13.1Jα41), and aaa10npa22 (Vα26.1Jα40) were expressed in duplicate. Remaining α-chain transcripts were unique when compared to each other and are shown in Table 4 (three clones) and S4 Table (15 clones).

The Vα6.1Jα53 clonal expansion, identified by NPA-PCR/Vα-specific PCR amplification was confirmed by Vα6-specific PCR followed by cloning and sequencing (Table 4). Four of 21 identical TCR transcripts (19%)(p = 0.01)(clone aaa10npa03)(Vα6.1Jα53)(CDR3:TPPG) identified by Vα6-specific PCR, cloning and sequencing, were identical to clone aaa10npa03, Vα6.1Jα53, CDR3:TPPG, identified by NPA-PCR/Vα-specific PCR (3 of 30 identical transcripts) (Table 4 and S4 Table).

Vα6-specific PCR amplification, cloning and sequencing showed an additional clonal expansion of 4 of 21 identical TCR transcripts (19%)(p = 0.01)(clone aaa10npa29)(Vα6.1Jα49) (CDR3:EAYS)(Table 4). This aaa10npa29 clone was also identified after NPA-PCR/Vα-specific PCR (single transcript, 1 of 30). Three clones amplified by Vα6-specific PCR were expressed in duplicate and 7 clones were expressed in single copies (Table 4 and S4 Table).

Sequence analysis of α-chain TCR transcripts after NPA-PCR/Vα-specific PCR amplification and cloning from AAA lesions from patient AAA12, revealed 3 clones aaa12npa26 (Vα13.1Jα31), aaa12npa02 (Vα13.1Jα45) and aaa12npa16 (Vα26.1Jα45) expressed in duplicate (2 of 31; 6%; p = 0.19)(Table 4). Remaining 25 α-chain TCR transcripts were unique when compared to each other and are shown in Table 4 (three clones) and S5 Table (nineteen clones). Further analysis of TCR transcripts from patient AAA12 was not carried out.

Grossly normal autopsy specimens of infrarenal abdominal aortas from 3 patients who died of nonvascular diseases were used as controls. RNA was prepared and β-chain TCR NPA-PCR /Vβ-specific PCR revealed the absence of β-chain TCR transcripts and of infiltrating T cells in these non-aneurysmal aortic tissue specimens [41]. These findings are in agreement with the reports of others that T cells or CD45+ cells are absent in nonaneurysmal aortic tissue [7,72,73]. Along these lines, we have reported the absence of infiltrating T cells from central epicardial arteries [61].

### PBMC from normal donors (methodological controls) are comprised of polyclonal T cells

We employed PBMC from 3 normal donors as methodological controls to ensure that all methods used in this study were performing well, as expected. Sequence analysis of α-chain TCR transcripts after NPA-PCR/Vα-specific PCR and cloning demonstrated that these transcripts were productively rearranged human α-chain TCR transcripts and typical of polyclonal T cells (Table 5, normal donor 1) (S6 Table), in agreement with our previous findings [59,60]. A total of 170 α-chain TCR transcripts were sequenced from PBMC from normal donors (Table 5, S6 Table, and [59,60]) and all were unique when compared to each other with the exception of 12 of 170 transcripts (7%), which appeared in duplicate: (i) 2 of 28, normal donor 1 (Table 5); (ii) 2 of 47, normal donor 2 (S6 Table); (iii) 6 of 50, normal donor 3 (S6 Table); (iv) 1 of 25 [59]; and (v) 1 of 20 [60]. As it was mentioned in Methods (above), appearance in two different colonies of an identical α-chain TCR transcript, designated as a doublet, may indicate the beginning of a clonal expansion or may be the result of an artifact of the *E*. *coli* transfection method, and in particular of a single *E*. *coli* cell that was transformed and divided (doubled) before plating.

**Table 5:** α-chain TCR Transcripts (CDR3 Region) Identified in PBMC from Normal Donors.

Clone	Vα	N	Jα	Transcript Frequency in Specimen	p value	
**α-chain TCR transcripts from Normal Donor 1 amplified by NPA-PCR/Vα-specific PCR**					vs. 1/28	vs. 2/28
NBAnpa09	**C A M R**	**E G R V**	**G T A S K L**	Vα6.1Jα44	ns	ns
	tgtgcaatgaga	gagggccgtgt	cggcactgccagtaaactc	2/28		
NBAnpa12	**C A L**	**R G**	**L I K A A G N K L**	Vα12.1Jα17	ns	ns
	tgtgctctg	agggggt	tgatcaaagctgcaggcaacaagcta	1/28		
NBAnpa28	**C A V**	**N R**	**G Y Q K V**	Vα2.1Jα13	ns	ns
	tgtgccgtg	aata	ggggttaccagaaagtt	1/28		
NBAnpa02	**C A T**	**E G V**	**D Y K L**	Vα3.1Jα20	ns	ns
	tgtgctacg	gagggggt	cgactacaagctc	1/28		
NBAnpa16	**C A M R**	**A G P**	**G T A L**	Vα6.1Jα15	ns	ns
	tgtgcaatgaga	gccggtc	caggaactgctctg	1/28		
NBAnpa07	**C A M R**	**E G G**	**D N Y G Q N F**	Vα6.1Jα26	ns	ns
	tgtgcaatgaga	gagggcg	gggataactatggtcagaatttt	1/28		
NBAnpa36	**C A L**	**K A**	**G G S Y I P**	Vα9.1Jα6	ns	ns
	tgtgctcta	aagg	caggaggaagctacatacct	1/28		
NBAnpa05	**C A G**	**A V P K**	**Y G N K L**	Vα10.1Jα47	ns	ns
	tgtgcagga	gctgtcccca	aatatggaaacaaactg	1/28		
NBAnpa03	**C A L S**	**F**	**N A G N N R K L**	Vα12.1Jα38	ns	ns
	tgtgctctgagt	tt	taatgctggcaacaaccgtaagctg	1/28		
NBAnpa34	**C A V**	**T T**	**T G A N S K L**	Vα13.1Jα56	ns	ns
	tgtgctgtg	acaacg	actggagccaatagtaagctg	1/28		
NBAnpa23	**C A V**	**T R**	**T G A N I K L**	Vα13.1Jα56	ns	ns
	tgtgctgtg	acacga	actggagccaatattaagctg	1/28		
NBAnpa08	**C A E S**	**I S**	**S S A S K I**	Vα15.1Jα3	ns	ns
	tgtgcagagagt	ataag	cagcagtgcttccaagata	1/28		
NBAnpa27	**C A E S**	**S L**	**N T G G F K T**	Vα15.1Jα9	ns	ns
	tgtgcagagagt	tccctt	aatactggaggcttcaaaact	1/28		
NBAnpa22	**C A E**	**L N**	**Q A G T A L**	Vα15.1Jα15	ns	ns
	tgtgcagag	ctaa	accaggcaggaactgctctg	1/28		
NBAnpa21	**C A E S**	**M T**	**A A G N K L**	Vα15.1Jα17	ns	ns
	tgtgcagagagt	atgacg	gctgcaggcaacaagcta	1/28		
NBAnpa32	**C A A S**	**R M**	**D S N Y Q L**	Vα17.1Jα33	ns	ns
	tgtgcagcaagc	agaa	tggatagcaactatcagtta	1/28		
NBAnpa37	**C A V**	**R Y L G G**	**G A T N K L**	Vα19.1Jα32	ns	ns
	tgtgctgtc	agatatttagggggt	ggtgctacaaacaagctc	1/28		
NBAnpa29	**C A V**	**V M**	**Y G N K L**	Vα19.1Jα47	ns	ns
	tgtgctgtc	gtgatg	tatggaaacaagctg	1/28		
NBAnpa25	**C L V**	**G P F**	**N N A R L**	Vα20.1Jα31	ns	ns
	tgcctcgtg	ggtccctt	taacaatgccagactc	1/28		
NBAnpa15	**C A G**	**Q L D**	**N T D K L**	Vα25.1Jα34	ns	ns
	tgtgctggg	cagctgg	ataacaccgacaagctc	1/28		
NBAnpa20	**C A G**	**L**	**S G T Y K Y**	Vα25.1Jα40	ns	ns
	tgtgctggt	ct	ctcaggaacctacaaatac	1/28		
NBAnpa35	**C A G**	**P R**	**T G T A S K L**	Vα25.1Jα44	ns	ns
	tgtgctggg	ccgagg	accggcactgccagtaaactc	1/28		
NBAnpa33	**C G A D**	**R G**	**D S S Y K L**	Vα26.1Jα12	ns	ns
	tgtggagcagac	cgagg	ggatagcagctataaattg	1/28		
NBAnpa30	**C G**	**P L V P H**	**S G G G A D G L**	Vα26.1Jα45	ns	ns
	tgtgga	cccctcgtacctc	attcaggaggaggtgctgacggactc	1/28		
NBAnpa10	**C R N L**	**L L H M**	**D T G R R A L**	Vα30.1Jα5	ns	ns
	tgtaggaaccta	ctcctccaca	tggacacgggcaggagagcactt	1/28		
NBAnpa11	**C L L G**	**S T F Y**	**N N N D M**	Vα31.1Jα43	ns	ns
	tgtcttctggga	tctaccttct	acaataacaatgacatg	1/28		
**Results from normal donors 2 and 3 are shown in the supporting information section S6 Table.**						

### Control studies using PBMC from normal donors demonstrate that these results reveal true clonal expansions of T cells

The clonal expansions reported here were obtained by amplifying TCR transcripts by two PCR methods. An argument could be made that in the event that these two PCR amplifications were performed only from very few T cells, then it could be possible that each pair of amplification primers would amplify TCR transcripts from only few T cells, yielding findings that could resemble those shown in this paper. We have carried out extensive control experiments [41,58,59,62,63] using β-chain TCR transcripts demonstrating that this is not the case, that these results are true clonal expansions of T cells and they are not due to PCR amplification of TCR transcripts from just a few numbers of T cells. These results have been presented elsewhere [41,58,59,62,63] and will be briefly discussed here to address this point.

As it was mentioned above, each specimen containing AAA lesions from patients with AAA was divided into two halves. One half was employed for immunohistochemistry and the other half for RNA preparation, and TCR amplification, cloning and sequencing. The yield of RNA was approximately 10 μg per preparation, which represents approximately 1.0 x 107 cells. An amount of 50 ng of RNA was used for each PCR amplification, cloning and sequencing. It is estimated that these 50 ng of RNA are isolated from approximately 5.0 x 104 cells.

The representation (ratio) of different TCR clones is the same in a sample of 10 μg RNA and a sample of 50 ng RNA that we employed for PCR, cloning, and sequencing. The clonally expanded TCR transcripts that were identified in 10 μg RNA, are also present in 50 ng RNA; the ratio of the different clonally expanded TCR transcripts to each other does not change. However, when different amounts of RNA are used in the PCR amplification the absolute number of copies of the clonally expanded TCR transcripts that are present is different.

We employed an anti-CD3 mab and immunohistochemical staining to determine the numbers of CD3+ T lymphocytes present in AAA specimens that we used for RNA preparation from patients AAA09 and AAA10. CD3+ T cells were counted in a large number (twenty) of high power fields per specimen by two different observers, independently. CD3+ T cell numbers varied substantially (range 0 to 155 CD3+ T lymphocytes) among each high power field. An average number of CD3+ T lymphocytes of approximately 780 per section and 660 per section were found in specimens AAA09 and AAA10, respectively. Because the thickness of aorta tissue specimens was approximately 5 mm and the thickness of the cryostat sections of the aorta specimens used were 6 μm thick, the total number of CD3+ T lymphocytes employed for RNA isolation from aortic specimens from patients AAA09 and AAA10 were estimated to be 6.5x105 and 5.5x105, respectively. In consideration that 10 μg of RNA, derived from approximately 1x107 total cells present in these specimens, was recovered per preparation, CD3+ T lymphocytes alone in the AAA09 and AAA10 specimens were 6.5% and 5.5%, respectively (mean 6%), of the total cells employed to isolate RNA. An amount of 50 ng of RNA, representing approximately 5x104 cells, were employed for PCR amplification. CD3+ T lymphocytes accounted for about 6% of these cells, i.e., approximately 3,000 T cells.

Further control experiments were performed to identify the threshold of the minimum number of CD3+ T lymphocytes present in normal donor PBMCs that will provide polyclonal TCR transcripts after two PCR cycles, cloning and sequencing. Sequence analysis, after NPA-PCR amplification with various amounts of cDNA template and cloning, starting with as low as 300 T cells [41], demonstrated the presence of unique transcripts when compared with each other, except of two TCR transcripts which were present in duplicate (statistically not significant), typical of polyclonal T cells. The numbers of these T lymphocytes were 10 times lower than those present in AAA specimens used in these experiments (i.e., 3,000 T lymphocytes, as determined by immunohistochemistry using an anti-CD3 mab, see above).

The same approach was used in connection with Vβ-specific PCR, followed by cloning and sequencing [41,58,59,62,63]. Vβ2-specific PCR followed by cloning and sequencing of normal donor PBMC containing as low as 1,200 T cells and an estimated 100 Vβ2+ T lymphocytes (in 50 ng RNA), demonstrated unique Vβ2+ TCR transcripts when compared to each other with the exception of two TCR transcripts present in duplicate (not statistically significant), typical of polyclonal T lymphocyte populations. The T cell numbers, 1,200 T cells, used in these experiments were lower than those, 3,000 T cells, in 50 ng RNA from AAA specimens.

Sequence analysis after Vβ2-specific PCR amplification and cloning, from another mixture that contained only 300 T cells from the peripheral blood of normal donors, corresponding to 24 Vβ2+ T lymphocytes, showed a more restricted pattern, consisting of the following [41]: (i) one Vβ2+ transcript in triplet; (ii) 4 transcripts in duplicate copies; and (iii) 8 other transcripts in a single copy. These clonal expansions are not statistically significant. These findings confirm that the clonal expansions of T lymphocytes identified in AAA lesions represent real clonal expansions and are not due to amplifications of TCR transcripts from just a few T cells.

### DNA-based HLA-typing for HLA-DRB1, -DQA1 and -DQB1

Three of 5 patients with AAA were typed by DNA-based HLA-typing for HLA-DRB1, -DQA1 and -DQB1 (Table 6). All three patients, AAA03, AAA09 and AAA10, expressed DRB1 alleles positive for the DRβGln70 amino acid residue, which was reported to be associated with AAA [21]. Clonally expanded T cells in AAA lesions were present in all these 3 patients (Table 4). DNA-based HLA-typing of six patients with AAA, including the three patients shown here, has been reported previously [41], and is shown here to present a complete picture.

**Table 6:** DNA-based typing for HLA-DRB1, -DQA1 and -DQB1 loci of patients with AAA*.

Sample Name	DRB1-1	DRB1-2	DQA1-1	DQA1-2	DQB1-2	DQB1-2	DRβQ70
AAA03	0301	0101	0101	0501	0201	0501	+
AAA09	0101	0701	0101	0201	0202	0501	+
AAA10	0301	1501	0102	0501	0201	0602	+

* DNA-based HLA-typing results of these three patients with AAA have been reported previously [41] as part of a larger series and are shown here for the shake of completion. Reproduced in part, with the permission of The American Association of Immunologists, Inc., from Lu et al, J. Immunol., 192: 4897–4912, 2014 (reference [41]). Copyright 2014. The American Association of Immunologists, Inc.

### Conserved CDR3 amino acid motifs

A substantial number of CDR3 amino acid motifs were found in higher proportions in the TCR CDR3 from AAA lesions of patients with AAA vs. those of PBMC from normal donors, which were used as methodological controls (Table 7). These CDR3 amino acid motifs were selected with two amino acids each, because examination of the CDR3 sequences identified in AAA lesions revealed the presence of CDR3 motifs comprised of two amino acids each that were expressed in high proportions in more than one patients with AAA. The proportions of these CDR3 two amino acid motifs in patients with AAA were compared to the proportions of these CDR3 two amino acid motifs in PBMC from normal donors (Table 7) and were statistically significant by the differences in Poisson rates. The percent proportions of 17 of 28 CDR3 two amino acid motifs identified in AAA lesions were statistically significant in comparison to the percent proportions of these CDR3 two amino acid motifs expressed in PBMC from normal donors. We have previously used this approach to analyze CDR3 motifs [41]. Certain α-chain TCR CDR3 two amino acid motifs were found in increased proportions in more than one AAA patient (Table 7). Different α-chain TCR CDR3 amino acid motifs were utilized by different patients with AAA, suggesting the recognition of different peptide/MHC complexes by clonally expanded T cells or other T-cell clones present in AAA lesions from different patients. A total of 170 α-chain TCR transcripts from PBMC of 5 normal donors (Table 5 and [59,60]), were used as normal controls (Table 7).

**Table 7:** CDR3 α-chain TCR conserved amino acid motifs found in AAA lesions of patients with AAA.

CDR3 Motif	*AAA Patient*					
	AAA03	AAA09	AAA00	AAA10	AAA12	*Normal PBMC*
AG	26/99 (26%)	8/96 (8%)	0/30 (0%)	1/51 (2%)	2/31 (6%)	40/170 (23%)
AL	28/99 (28%)	48/96 (50%)	0/30 (0%)	2/51 (4%)	3/31 (10%)	25/170 (15%)
AY	6/99 (6%)	2/96 (2%)	0/30 (0%)	7/51 (14%)	0/31 (0%)	4/170 (2%)
EA	1/99 (1%)	2/96 (2%)	0/30 (0%)	6/51 (12%)	0/31 (0%)	2/170 (1%)
EE	4/99 (4%)	0/96 (0%)	15/30 (50%)	0/51 (0%)	6/31 (19%)	1/170 (0.5%)
EG	36/99 (36%)	11/96 (11%)	15/30 (50%)	2/51 (4%)	5/31 (16%)	9/170 (5%)
EP	0/99 (0%)	14/96 (15%)	0/30 (0%)	0/51 (0%)	0/31 (0%)	1/170 (0.5%)
GG	21/99 (21%)	23/96 (24%)	30/30 (100%)	30/51 (59%)	22/31 (71%)	42/170 (25%)
GGG	4/99 (4%)	2/96 (2%)	0/30 (0%)	8/51 (16%)	0/31 (0%)	8/170 (5%)
GR	18/99 (18%)	2/96 (2%)	0/30 (0%)	1/51 (2%)	0/31 (0%)	8/170 (5%)
GS	20/99(20%)	17/96 (18%)	15/30 (50%)	5/51 (10%)	3/31 (10%)	35/170 (20%)
GT	5/99 (5%)	6/96 (6%)	15/30 (50%)	14/51 (27%)	2/31 (6%)	16/170 (9%)
LE	25/99 (25%)	0/96 (0%)	0/30 (0%)	0/51 (0%)	0/31 (0%)	9/170 (5%)
PF	0/99 (0%)	14/96 (15%)	0/30 (0%)	3/51 (6%)	0/31 (0%)	52/170 (30%)
PP	0/99 (0%)	0/96 (0%)	0/30 (0%)	7/51 (14%)	2/31 (6%)	0/170 (0%)
RE	23/99 (23%)	16/96 (17%)	0/30 (0%)	14/51 (27%)	1/31 (3%)	3/170 (2%)
RG	0/99 (0%)	18/96 (19%)	0/30 (0%)	0/51 (0%)	3/31 (10%)	2/170 (1%)
SA	39/99 (39%)	10/96 (10%)	0/30 (0%)	0/51 (0%)	3/31 (10%)	6/170 (3%)
SE	0/99 (0%)	20/96 (21%)	0/30 (0%)	0/51 (0%)	0/31 (0%)	14/170 (8%)
SG	17/99 (17%)	19/96 (20%)	0/30 (0%)	18/51 (35%)	4/31 (13%)	32/170 (19%)
SS	35/99 (35%)	8/96 (8%)	0/30 (0%)	3/51 (6%)	5/31 (16%)	7/170 (4%)
SY	10/99 (10%)	4/96 (4%)	0/30 (0%)	10/51 (20%)	3/31 (10%)	29/170 (17%)
TG	7/99 (7%)	24/96 (25%)	30/30 (100%)*	6/51 (12%)	0/31 (0%)	9/170 (5%)
TP	1/99 (1%)	1/96 (1%)	0/30 (0%)	8/51 (16%)	0/31 (0%)	20/170 (12%)
VS	24/99 (24%)	1/96 (1%)	0/30 (0%)	0/51 (0%)	5/31 (16%)	27/170 (16%)
VT	2/99 (2%)	1/96 (1%)	15/30 (50%)	1/51 (2%)	1/31 (3%)	2/170 (1%)
VV	28/99 (28%)	1/96 (1%)	15/30 (50%)	0/51 (0%)	5/31 (16%)	13/170 (8%)
YS	26/99 (26%)	6/96 (6%)	0/30 (0%)	8/51 (16%)	0/31 (0%)	5/170 (3%)


*Each one of 15 transcripts expressed 2 TG amino acid motifs (30 transcripts were sequenced from patient AAA00).

Statistical Analysis: **AG**: AAA vs. Normal PBMC, **p = 0.0034**; **AL**: AAA vs. Normal PBMC, **p = 0.0106**; AY: AAA vs. Normal PBMC, p = 0.1941; EA: AAA vs. Normal PBMC, p = 0.2398; **EE**: AAA vs. Normal PBMC, **p = 0.0100238**; **EG**: AAA vs. Normal PBMC, **p<0.0001**; **EP**: AAA vs. Normal PBMC, **p = 0.0479**; **GG**: AAA vs. Normal PBMC, **p = 0.0044**; GGG: AAA vs. Normal PBMC, p = 0.9435; GR: AAA vs. Normal PBMC, p = 0.3680; GS: AAA vs. Normal PBMC, p = 0.8067; GT: AAA vs. Normal PBMC, p = 0.2030; LE: AAA vs. Normal PBMC, p = 0.2680; **PF**: AAA vs. Normal PBMC, **p<0.0001**; PP: AAA vs. Normal PBMC, p = 0.1276; **RE**: AAA vs. Normal PBMC, **p = 0.0001**; **RG**: AAA vs. Normal PBMC, **p = 0.0174**; **SA**: AAA vs. Normal PBMC, **p = 0.0002**; SE: AAA vs. Normal PBMC, p = 0.5012; SG: AAA vs. Normal PBMC, p = 0.9867; **SS**: AAA vs. Normal PBMC, **p = 0.0005**; **SY**: AAA vs. Normal PBMC, **p = 0.0132**; **TG**: AAA vs. Normal PBMC, **p<0.0001**; **TP**: AAA vs. Normal PBMC, **p = 0.0009**; VS: AAA vs. Normal PBMC, p = 0.0671; **VT**: AAA vs. Normal PBMC, **p = 0.0210**; **VV**: AAA vs. Normal PBMC, **p = 0.0183; YS**: AAA vs. Normal PBMC, **p = 0.0017**.

### Comparison of the nucleic acid and deduced amino acid sequences of α-chain TCR transcripts to those in the GENBANK/EMBL database

Comparison of all α-chain TCR sequences identified here to those in the GenBank/EMBL database by the BLAST software showed that the α-chain TCR transcripts identified in this study were novel and typical of α-chain TCR. None of the α-chain TCR transcripts reported here were reported previously. Identical a-chain TCR transcripts were not found in AAA lesions from different AAA patients. However, comparison of the CDR3 motifs of the clonally expanded α-chain TCR transcripts in AAA lesions by the gapped BLAST and PSI BLAST protein database programs revealed certain highly homologous CDR3s between α-chain TCR transcripts that were clonally expanded in AAA lesions to those previously reported in the GenBank/EMBL. Currently, it has not been established what is the maximum number of CDR3 amino acid differences that defines high or extensive CDR3 homology. We have chosen arbitrarily that differences of two conservative and one non-conservative amino acids would be the maximum number of differences allowed between CDR3 regions from different T-cell clones in order to define high or extensive CDR3 homology. Most important homologies include:

**Patient AAA03** (Table 4): Clonally expanded clone aaa03npa04, CDR3: CALEGYSSASKI, exhibited extensive CDR3 homology with 3 T-cell clones: (i) CDR3: CALASYSSASKI, GenBank Accession No. ANO54287.1; (ii) CDR3: CALSYSSASKI, GenBank Accession No. ANO55176.1; (iii) CDR3: CAEKRGYSSASKI, GenBank Accession No. ANO54594.1; all identified in peripheral blood T cells of patients with Sjögren’s syndrome (SS) [74]. Clone aaa03npa04 showed extensive CDR3 homology with a T-cell clone, CDR3: CAAPGYSNASKI, GenBank Accession No. AAA80058.1, found on anti-DNA antibody-helper T cells from the peripheral blood of a lupus patient [75]. Clone aaa03npa33, CDR3: CAMREGEGGSNYKL, showed high CDR3 homology with a T-cell clone, CDR3: CAVKEAGG GSNYKL, GenBank Accession No. ANO55390.1 found in peripheral blood T cells of an SS patient [74].

Clonally expanded clone aaa03npa02, CDR3: CAGQKGGTSYGKL, exhibited extensive CDR3 homology with 5 T-cell clones: (i) CDR3: CAAQGGTSYGKL, GenBank Accession No. ANO54008.1; (ii) CDR3: CAANAGGTSYGKL, GenBank Accession No. ANO55694.1; both identified in SG of patients with SS [74]; (iii) CDR3: CAGRNAGGTSYGKL, GenBank Accession No. ANO54097.1; (iv) CDR3: CAENGGTSYGKL, GenBank Accession No. ANO54594.1; (v) CDR3: CAGAPAGGTSYGKL, GenBank Accession No. ANO54605.1; all 3 identified in peripheral blood of patients with SS [74].

Clone aaa03npa16, CDR3: CAVEETSGSRL, had extensive CDR3 homology with 5 T-cell clones: (i) CDR3: CAVVEETSGSRL, GenBank Accession No. ANO54242.1; (ii) CDR3: CAVRETSGSRL, GenBank Accession No. ANO56281.1; (iii) CDR3: CAVDRETSGSRL, GenBank Accession No. ANO56267.1; all 3 found in salivary glands (SG) of SS patients [74]; (iv) CDR3: CAVKETSGSRL, GenBank Accession No. BAF94397.1, human T-cell clone [76]; (v) CDR3: CAVRE TSGSRL, GenBank Accession No. AAB 97020.1; a human T-cell clone [77]. Clone aaa03va2408, CDR3: CVAATGGFKT, had extensive CDR3 homology with 3 T-cell clones: (i) CDR3: CAASTGGFKT, GenBank Accession No. ANO55378.1; (ii) CDR3: VADTGGFKT, GenBank Accession No. ANO 54837.1; both identified in SGs of SS patients [74]; (iii) CDR3: CAASTGGFKT, GenBank Accession No. AAC72697.1, human T-cell clone from the synovial fluid of a patient with rheumatoid arthritis [78].

**Patient AAA09** (Table 4): Clonally expanded clone aaa10npa08, CDR3: CALSEPFQTGANNL, had extensive CDR3 homology with a T-cell clone, CDR3: EYAQTGANNL, GenBank Accession No. ANO56562.1, found in SG of an SS patient [74]. Clone aaa09npa16, CDR3: CALSRGSQGNL, showed extensive CDR3 homology with 5 T-cell clones: (i) CDR3: CALVRGSQGNL, GenBank Accession No. ANO56100.1; (ii) CDR3: CAMRDSRGSQGNL, GenBank Accession No. ANO55703.1; (iii) CDR3: CAVRRGSQGNL, GenBank Accession No. ANO55251.1; all 3 found in SGs of SS patients [74]; (iv) CDR3: CALGRNYGGSQGNL, GenBank Accession No. ANO54721.1; identified in peripheral blood T cells of an SS patient [74]; (v) CDR3: CAVIGRGSQGNL, GenBank Accession No. AAA80100.1; found on an anti-DNA antibody helper T-cell clone from peripheral blood of a lupus patient [75]; (vi) CDR3: CALSVGSQGNL, GenBank Accession No. AIE10490.1; found in a human T-cell clone [79]. Clone aaa 09npa37, CDR3: CAMREIRTSYDKV, had extensive CDR3 homology with a T-cell clone, CDR3: CAM REYPSYDKV, GenBank Accession No. ABO16436.1; found in a patient with renal cell carcinoma [80].

**Patient AAA00** (Table 4): Clonally expanded clone aaa00va0201, CDR3: CVVTGTGGFKT, had extensive CDR3 homology with 3 T-cell clones: (i) CDR3: CVVNGAGGFKT, GenBank Accession No. ANO55862.1; (ii) CDR3: CVVSDGTGGFKT, GenBank Accession No. ANO56340.1; both identified in SGs of SS patients [74]; (iii) CDR3: CVVSEGTGGFKT, GenBank Accession No. ANO54058.1; found in peripheral blood T cells of an SS patient [74]. Clonally expanded clone aaa00va0801, CDR3: CAEEGGSNYKL, exhibited extensive CDR3 homology with: (i) CDR3: CAENRSGGSNYKL, GenBank Accession No. ANO55004.1; (ii) CDR3: CAENSSGGSNYKL GenBank Accession No. ANO55130.1; both identified in peripheral blood of an SS patient [74].

**Patient AAA10** (Table 4): Clonally expanded clone aaa10npa03, CDR3:CAMTPPGGGTSYGK had extensive CDR3 homology with a T-cell clone, CDR3: CAVSGPPAGGTSYGKL, GenBank Accession No. ANO54194; found in peripheral blood T cells of an SS patient [74]. Clone aaa10npa29, CDR3: CAMREAYSGNQFY, had extensive CDR3 homology with a T-cell clone, CDR3: CALSEANT GNQFY, GenBank Accession No. ANO55358; found in peripheral blood T cells of an SS patient [74].

A substantial number of clonally expanded α-chain TCR clones found in AAA lesions from patients with AAA, exhibited extensive CDR3 homology to T-cell clones found in SS patients.

#### Clonally expanded α- and β-chain TCR transcripts in AAA lesions of patients with AAA

Four of 5 patients who demonstrated in this study statistical significant clonal expansions of α-chain TCR transcripts in AAA lesions, also exhibited statistically significant clonal expansions of β-chain TCR transcripts. These results have been published previously [41] and are summarized here (Tables 8 and 9) for comparison purposes. In contrast, patient AAA12 exhibited polyclonal α- (Tables 4, 8 and 9) and β-chain [41] TCR transcripts in AAA lesions.

**Table 8:** TCR Clonal Expansions and Oligoclonality of T Cells Infiltrating AAA Lesions.

Patients with AAA	Clonal Expansion/Oligoclonality	
	α-chain	β-chain
AAA03	**Yes**	**Yes**
AAA09	**Yes**	**Yes**
AAA00	**Yes**	**Yes**
AAA10	**Yes**	**Yes**
AAA12	Polyclonal	Polyclonal

**Table 9:** Major Clonal Expansions of α- and β-chain TCR transcripts in AAA lesions.

Patient	α-chain % identical transcripts				β-chain % identical transcripts [41]			
AAA09	Vα12.Jα36	11/20	55%	p<0.0001*	Vβ14.1Dβ2.1Jβ2.3	12/21	57%	p<0.0001**
AAA00	Vα2.3Jα9 Vα8.2Jα54	15/15 15/15	100% 100%	p<0.0001* p<0.0001*	Vβ5.1Dβ2.1Jβ2.3	6/34	17.6%	p = 0.0004^+^
AAA03	Vα5.1Jα3 Vα24.1Jα47	20/20 13/22	100% 59%	p<0.0001* p<0.0001*	Vβ24.1Dβ2.1β1.3 Vβ6.3Dβ2.1Jβ2.1 Vβ3.1Dβ2.1Jβ2.1	17/20 4/21 5/24	85% 19% 21%	p<0.0001** p = 0.01**p = 0.002**
AAA10	Vα6.1Jα53 Vα6.1Jα49	4/21 4/21	19% 19%	p = 0.01* p = 0.01*	Vβ3.1Dβ2.1Jβ2.1 Vβ3.1Dβ1.1Jβ1.5	10/41 6/41	24% 15%	p<0.0001** p = 0.0004**
AAA12	Not significant				Not significant^++^			

* Clonally expanded α-chain TCR transcripts following Vα-specific PCR amplification, cloning and sequencing (this report).

**Clonally expanded β-chain TCR transcripts following Vβ-specific PCR amplification, cloning and sequencing [41].

+Clonally expanded β-chain TCR transcripts following NPA-PCR/Vβ-specific PCR amplification, cloning and sequencing [41].

++ Following NPA-PCR/Vβ-specific PCR amplification, cloning and sequencing [41].

## Discussion

To determine whether clonally-expanded α-chain TCR transcripts are present in T cells infiltrating AAA lesions from patients with AAA, we amplified α-chain TCR transcripts from these lesions by NPA-PCR/Vα-specific PCR and cloned and sequenced the amplified transcripts. Analysis of the sequences demonstrated the presence of high proportions of identical α-chain TCR transcripts in 4 of 5 patients with AAA. These results were confirmed by two-sided Vα-specific PCR, a independent amplification method, cloning and sequencing. Identical α-chain TCR clonal expansions to those obtained after NPA-PCR/Vα-specific PCR were found. These results provide an important confirmation of the presence of oligoclonal T cells in AAA lesions and strongly support the view that AAA is a specific antigen-driven T-cell disease.

Previously, we reported that that β-chain TCR transcripts are clonally expanded in AAA lesions from 8 of 10 patients with AAA [41]. In this study, we demonstrate that α-chain TCR transcripts in AAA lesions are also clonally expanded. Four of 5 patients with AAA who exhibited statistically significant α-chain TCR clonal expansions in AAA lesions, also exhibited statistically significant β-chain TCR clonal expansions [41] (Tables 8 & 9). AAA lesions from patient AAA12 contained polyclonal α- and β-chain TCR transcripts (Tables 8 & 9). In the studies reported here we have continued the testing of the hypothesis that T cells infiltrating AAA lesions are oligoclonal and AAA is a specific antigen(s)-driven T-cell disease. To complete the testing of this hypothesis additional studies will be needed to identify the antigen(s), self or non-self, recognized by the clonally expanded α- and β-chain TCR.

Identical α-chain TCR transcripts were not identified in AAA lesions from different patients with AAA. This is in agreement with our results [41,57–63,80,81] and those of others [74–78,82] in a large number of studies demonstrating the absence of sharing among different patients with the same disease of clonally expanded identical α- and β-chain TCR transcripts. Similarly, entire CDR3 segments were not shared among different patients [41,57–63,74–78,80,81]. There is extensive promiscuity in the interactions of TCR with peptide/MHC and a number of reasons may be responsible (reviewed in [80]): (i) Different T-cell clones expressing different TCR recognize peptides bound to different MHC class I or II alleles. Different peptides from the same antigens may be presented to T cells; (ii) Different antigenic peptides bind to the same MHC allele and these different peptide:MHC complexes may be recognized by different TCR; (iii) Different epitopes of a single peptide:MHC complex may be recognized by different TCR; (iv) The same peptide:MHC epitope may be recognized by several different TCR; (v) Several amino acids of the CDR3 are TCR clone specific and may be coded, at least in part, by random additions of N-nucleotide (nontemplated nucleotides) during the generation of T-cell diversity and not by nucleotides belonging to V, D (β-chain only) or J gene segments [74]. However, a substantial number of CDR3 amino acid motifs, comprised of two amino acids each, were expressed in higher proportions (statistically significant) in the CDR3 of several T-cell clones from AAA lesions in a number of patients vs. those of PBMC from normal donors, used as methodological controls (Table 5). These CDR3 two amino acid motifs, may be coded by nontemplated nucleotides, (see above, [74]), and may have undergone selection by antigenic stimulation. Preferential Vβ22 and Vβ25 utilization was found [83] in aneurysmic lesions from 10 of 14 patients with Marfan Syndrome, familial thoracic aortic aneurisms (TAAs) and patients with sporadic TAAs.

We studied here TCR transcripts from fresh (uncultured) T cells, and not T cells expanded in vitro, in culture with recombinant IL-2 (rIL-2). T-cell lines expanded in culture with rIL-2 are comprised of different proportions of T-cell clones vs. those present in fresh, uncultured, T cells from the same donor, and exhibit different properties such as cytokine production [84]. Growth rates of different T-cell clones in culture with rIL-2 are often different. Expansion of T cells in different concentrations of rIL-2 yields T-cell lines with quite different properties [85].

The αβ TCR+ T-cell repertoire is very large [44–46,62,63] and the maximum theoretical number is 1018 different α/β TCR, 107 α-chain, and 1012 β-chain TCR transcripts [62]. Each T-cell clone is identified by a unique TCR and recognizes a different antigenic epitope (peptide plus MHC) through its TCR, which is the unique fingerprint of that particular T-cell clone. The number of T-cell clones is greatly reduced during thymic selection and only a small proportion survive and become mature T cells. Arstila et al [44] estimated that 1x106 different β-chain TCR transcripts may be expressed in different T-cell clones in PBMC of normal donors. Each one of them may be pairing with 25 or more different α-chain TCR [44]. Warren et al [63] used high-throughput sequencing of β-chain TCR to measure at least 1x106 distinct β-chain TCR in PBMC. Robins et al [45] used deep sequencing and a Poisson statistical model to estimate 3-4x106 β-chain TCR sequences in the peripheral blood, a 4-fold higher than the other two estimates. Qi et al [46] employed next-generation sequencing and non-parametric statistics to estimate the size of the β-chain TCR repertoire to be 100x106 β-chain TCR sequences in the peripheral blood. The size of the T-cell repertoire is very large and the number of different T-cell clones is very high and able to recognize all conceivable antigenic epitopes. Therefore, the probability is very small of finding by chance two or multiple identical α- or β-chain TCR transcripts in an independent sample of T cells. The presence of multiple identical copies of α- or β-chain TCR transcripts has to be the result of specific antigen-driven proliferation and clonal expansion of particular T-cell clone(s) in vivo in AAA lesions, in response to as yet unidentified antigen(s), self or non-self, that they recognize.

Several lines of evidence (Table 1), strongly suggest that autoimmunity may be responsible for the pathogenesis of AAA, which may be an autoimmune disease [3–5, 9–41]. The identification of the three components of the trimolecular complex in AAA, and in particular the clonal expansions of the α- and β-chain TCR of T cells in AAA lesions ([10, 41], and this study), the association of AAA with MHC Class I and II [20–22] and the identification of putative AAA antigens [23–36], provide a compelling argument that AAA is a specific antigen-driven T-cell disease. AAA formation is controlled by cells, cytokines, and small molecules that inhibit inflammation (Table 1, [86]). Impaired immunoregulation may also play a role [38–40]. Chronic inflammation mechanisms in AAA are typical to those in autoimmune disease [87] and the immune response to tumors [88]. However, formation of transient follicles has been observed during the destabilization of atherosclerotic plaques [89] and it could be suggested that such a mechanism may be responsible for the presence of mononuclear cell infiltrates in AAA lesions and that the immune response identified in AAA may be secondary in the disease process. However, others emphasize the differences between AAA and atherosclerosis and the increasingly popular view of the autoimmune hypothesis as responsible for the etiology of AAA versus the atherogenic theory [90]. Nevertheless, our understanding of the pathogenesis of AAA is still limited.

AAA is associated with certain HLA class I (HLA-A2, HLA-B61) and class II (HLA-DerRB1*02, -DRB1*04) alleles [20–22,35]. Three (AAA03, AAA09 and AAA10) of the 5 patients studied here were typed by DNA-based HLA-typing approaches and had DRB1 alleles positive for the DRβGln70 amino acid residue (Table 4). These 3 patients exhibited in AAA lesions statistically significant clonal expansions of both α- and β-chain TCR transcripts. DNA-based HLA-typing of 6 patients with AAA [41], including the 3 patients shown here, revealed the expression of the DRβGln70 amino acid residue in 5 of 6 patients [41]. Clonally expanded β-chain TCR transcripts were found in 5 of these 6 patients and 4 expressed DRβGln70 [41]. The DRβGln70 amino acid residue is associated with AAA [21, 22] and it forms together with amino acid residues in positions 67, 71 and 74 a binding peptide pocket (#4) in HLA-DRB1 [91,92], which is associated with certain autoimmune disorders [91–93]. A large number of DRB1 alleles with Gln at position 70 of the β-chain have been identified [55].

Our studies demonstrate clonally expanded αβ TCR in AAA lesions and may permit identification of the three molecular components of the trimolecular complex, the αβ TCR, the HLA-DRβGln[70](and perhaps other HLA epitopes) and the AAA-associated antigens (peptides), self [3,18,19,23–26,30] and non-self [31–36], responsible for the immunopathogenesis of the disease. These antigens may be involved in the immunopathogenesis of AAA and in particular the initiation and/or the propagation of the disease. Molecular mimicry [37], which is defined as the sharing of cross-reactive antigenic determinants between host antigens and microorganisms, including viruses or bacteria, may be involved in the pathogenesis of AAA [35]. AAA may be initiated by an immune response to a virus or bacterium, which may crossreact with an antigenic epitope of a self-antigen, by molecular mimicry. After the clearance of the microorganism, the initial immune response may be propagated by the crossreactive antigenic epitope(s) of a self-antigen [37]. Molecular mimicry may be more likely responsible for the pathogenesis of the disease than purely autoreactive T cell clones. T cells with high affinity for self-antigenic determinants would have been eliminated during thymic selection. In contrast, T cell clones that recognize crossreactive antigenic determinants of host antigens and microorganisms may escape elimination during thymic selection. In addition to intiation, propagation/progression is very important for the development of clinical disease. The evidence suggesting that the immune system is responsible for propagation/progression of the disease is strong and it is further supported by the unique ability of the immune system to exhibit immunological memory.

Our results (this report and [10,41]) provide strong evidence supporting the hypothesis that AAA is a specific antigen-driven T cell disease. The identification of the clonally-expanded TCR transcripts in AAA lesions, may permit the identification of the antigens, self or nonself, recognized by the clonally expanded T lymphocytes. These AAA-associated antigens may play a critical role in the initiation and/or the propagation of the disease, and identification of their role is critical for understanding AAA and may permit the development of new therapies for the management of aneurismal disease.

## Supporting information

S1 Table Human α-chain TCR primers used for amplification. (DOCX)

S2 Table Additional α-chain TCR Transcripts (CDR3 Region) to those shown in Table 4, Expressed in the Aneurysmal Wall of Patient AAA03. These α-chain TCR transcripts were unique when compared to each other.
(DOCX)

S3 Table Additional α-chain TCR Transcripts (CDR3 Region) to those shown in Table 4, Expressed in the Aneurysmal Wall of Patient AAA09. These α-chain TCR transcripts were unique when compared to each other.
(DOCX)

S4 Table Additional α-chain TCR Transcripts (CDR3 Region) to those shown in Table 4, Expressed in the Aneurysmal Wall of Patient AAA10. These α-chain TCR transcripts were unique when compared to each other.
(DOCX)

S5 Table Additional α-chain TCR Transcripts (CDR3 Region) to those shown in Table 4, Expressed in the Aneurysmal Wall of Patient AAA12. These α-chain TCR transcripts were unique when compared to each other.
(DOCX)

S6 Table Additional α-chain TCR Transcripts (CDR3 Region) to those shown in Table 5, Identified in PBMC from Normal Donors. These α-chain TCR transcripts were unique when compated to each other.
(DOCX)
