# Supplementary material for: Clonally expanded alpha-chain T-cell receptor (TCR) transcripts are present in aneurysmal lesions of patients with Abdominal Aortic Aneurysm (AAA)
Source: PLoS One. 2019 Jul 16;14(7):e0218990. doi: 10.1371/journal.pone.0218990 (PMC6634378; doi:10.1371/journal.pone.0218990)
Supplement: S1 Table — (DOCX) [file pone.0218990.s001.docx]

**SUPPORTING INFORMATION**

**S1 Table: Human α-chain TCR Primers Used for Amplification**

| Primer | Sequence |
| --- | --- |
| **NPA-PCR** |  |
| **5’ end primer:** |  |
| Adaptor | AATTCGAACCCCTTCGAGAATGCG |
| **3’ end primer:** |  |
| Anti-adaptor | pCGCATTCTCGAAGGGGTTCG |
| hCα3 | CTGGATTTAGGTCTCTCAGCTGGTAC |
| hCα2 | GATATACACATCAGAATCCTTAC |
| hCα1 | GTTGCTCCAGGCCACAGCACTG |
|  |  |
| **Vα-specific amplifications** |  |
| **5’ end primer:** |  |
| Vα1_sp_5’ | CTGAGGTGCAACTACTCA |
| Vα2_sp_5’ | CAGTGTTCCAGAGGGAGCCA |
| Vα3_sp_5’ | GGTGAACAGTCAACAGGGAGA |
| Vα4_sp_5’ | TTGGTATCGACAGCTTCCCTCCCA |
| Vα5_sp_5’ | GGCCCTGAACATTCAGGA |
| Vα6_sp_5’ | GTCACTTTCTAGCCTGCTGA |
| Vα7_sp_5’ | GCAACATGCTGGCGGAGCACCCAC |
| Vα8_sp_5’ | CATTCGTTCAAATGTGG |
| Vα9_sp_5’ | ATCTCAGTGCTTGTGATAATA |
| Vα10_sp_5’ | ACCCAGCTGCTGGAGCAGAGCCCT |
| Vα11_sp_5’ | AGAAAGCAAGGACCAAGTGTT |
| Vα12_sp_5’ | CAGAAGGTAACTCAAGCGCAGACT |
| Vα13_sp_5’ | GAGCCAATTCCACGCTGCG |
| Vα14_sp_5’ | CAGTCTCAACCAGAGATGTC |
| Vα15_sp_5’ | GATGTGGAGCAGAGTCTTTTC |
| Vα16_sp_5’ | TCAACGTTGCTGAAGGGAATCCTC |
| Vα17_sp_5’ | GCTTATGAGAACACTGCGT |
| Vα18_sp_5’ | GCAGCTTCCCTTCCAGCAAT |
| Vα19_sp_5’ | AGAACCTGACTGCCCAGGAA |
| Vα20_sp_5’ | CATCTCCATGGACTCATATGA |
| Vα21_sp_5’ | GACTATACTAACAGCATGT |
| Vα22_sp_5’ | TACACAGCCACAGGATACCCTTCC |
| Vα23_sp_5’ | TGCCTCGCTGGATAAATCATCAGG |
| Vα24_sp_5’ | GGAGGGAAAGAACTGCACTCTT |
| Vα25_sp_5’ | ATCAGAGTCCTCAATCTATGTTTA |
| Vα26_sp_5’ | AGAGGGAAAGAATCTCACCATAA |
| Vα27_sp_5’ | ACCCTCTGTTCCTGAGCATG |
| Vα28_sp_5’ | CAAAGCCCTCTATCTCTGGTT |
| Vα29_sp_5’ | AGGGGAAGATGCTGTCA |
| Vα30_sp_5’ | CTTCACCCTGTATTCAGCTGGG |
| Vα31_sp_5’ | CTGCAGCTTCTTCAGAGAGAGACAATGG |
| Vα32_sp_5’ | TGCAAATTCCTCAGTACCAGCA |
|  |  |
| **3’ end primer:** |  |
| hCα2 | GATATACACATCAGAATCCTTAC |
