# Supplementary material for: Clonally expanded alpha-chain T-cell receptor (TCR) transcripts are present in aneurysmal lesions of patients with Abdominal Aortic Aneurysm (AAA)
Source: PLoS One. 2019 Jul 16;14(7):e0218990. doi: 10.1371/journal.pone.0218990 (PMC6634378; doi:10.1371/journal.pone.0218990)
Supplement: S2 Table — These α-chain TCR transcripts were unique when compared to each other. (DOCX) [file pone.0218990.s002.docx]

**S2 Table: Additional α-chain TCR Transcripts (CDR3 Region) to those shown in Table2, Expressed in the Aneurysmal Wall of Patient AAA03. These alpha-chain TCR transcripts were unique when compared to each other.**

| **Clone** | | | **Vα N Jα** | | **Transcript Frequency in Specimen** | **p value** | | | | | |  |  |
| --- | --- | --- | --- | --- | --- | --- | --- | --- | --- | --- | --- | --- | --- |
| **α-chain TCR transcripts amplified by NPA-PCR/Vα-specific PCR** | | | | | | **vs.**  **1/57** | | | **vs.**  **2/57** | | |  |  |
| aaa03npa63 | | | **C A M R E A Y S S A S K I**  tgtgcaatgaga gaggc gtacagcagtgcttccaagata | | Vα6.1Jα3  1/57(2%) | ns | | | ns | | |  |  |
| aaa03npa31 | | | **C A L G S G G G N K L**  tgtgctcta ggtt cgggaggaggaaacaaactc | | Vα9.1Jα10  1/57(2%) | ns | | | ns | | |  |  |
| aaa03npa28 | | | **C A V E E R Q A G T A L**  tgtgctgtggag gagagg caggcaggaactgctctg | | Vα11.1Jα15  1/57(2%) | ns | | | ns | | |  |  |
| aaa03npa15 | | | **C A P E G N T P L**  tgtgcc ccagag ggaaacacacctctt | | Vα11.1Jα29  1/57(2%) | ns | | | ns | | |  |  |
| aaa03npa71 | | | **C A V E D I D R L**  tgtgctgtggag gatatcga cagactc | | Vα11.1Jα31  1/57(2%) | ns | | | ns | | |  |  |
| aaa03npa08 | | | **C A L I S N A R L**  tgtgctctg atcagt aatgccagactc | | Vα12.1Jα31  1/57(2%) | ns | | | ns | | |  |  |
| aaa03npa24 | | | **G V Y L I G T K L**  ggcgtttat tt aattggaacaaagctc | | Vα13.1Jα13  1/57(2%) | ns | | | ns | | |  |  |
| aaa03npa69 | | | **C A A G F S D G Q K L**  tgtgct gcgggc ttttcagatggccagaagctg | | Vα13.1Jα15  1/57(2%) | ns | | | ns | | |  |  |
| aaa03npa54 | | | **C A Y I S G N K L**  tgtgcttat atc tctggaaacaaactg | | Vα14.1Jα47  1/57(2%) | ns | | | ns | | |  |  |
| aaa03npa30 | | | **C A F M K G G G N K L**  tgtgctttcatg aaaggcggc ggaaacaagctg | | Vα14.2Jα47  1/57(2%) | ns | | | ns | | |  |  |
| aaa03npa58 | | | **C A E S M A G S G G Q K L**  tgtgcagagagt atggccgggagtgga ggccagaagctg | | Vα15.1Jα15  1/57(2%) | ns | | | ns | | |  |  |
| aaa03npa05 | | | **C A E T Y K G K L**  tgtgcagag acctataaggg caagctc | | Vα15.1Jα34  1/57(2%) | ns | | | ns | | |  |  |
| aaa03npa13 | | | **C A F N N A G N M L**  tgtgcc tt taataatgcaggcaacatgctc | | Vα18.1Jα39  1/57(2%) | | | ns | | | ns | | |
| aaa03npa41 | | | **C A V G Y N N D M**  tgtgctgtc ggtt ataacaatgacatg | | Vα19.1Jα43  1/57(2%) | | | ns | | | ns | | |
| aaa03npa72 | | | **C A V L R P G N Q F**  tgtgctgtc ttaaggc ccggtaaccagttc | | Vα19.1Jα49  1/57(2%) | | | ns | | | ns | | |
| aaa03npa06 | | | **C A L G G A T N K L**  tgtgctctg ggt ggtgctacaaacaagctc | | Vα22.1Jα32  1/57(2%) | | | ns | | | ns | | |
| aaa03npa46 | | | **C V A Y N N N D M**  tgtgtt gctt acaataacaatgacatg | | Vα28.1Jα43  1/57(2%) | | | ns | | | ns | | |
| aaa03npa03 | | | **C A G P K S G G S N Y K L**  tgtgcaggg cctaaa agtggaggtagcaactataaactg | | Vα32.1Jα54  1/57(2%) | | | ns | | | ns | | |
|  | | | | | | |  | | |  | | |  |
