# Supplementary material for: Clonally expanded alpha-chain T-cell receptor (TCR) transcripts are present in aneurysmal lesions of patients with Abdominal Aortic Aneurysm (AAA)
Source: PLoS One. 2019 Jul 16;14(7):e0218990. doi: 10.1371/journal.pone.0218990 (PMC6634378; doi:10.1371/journal.pone.0218990)
Supplement: S3 Table — These α-chain TCR transcripts were unique when compared to each other. (DOCX) [file pone.0218990.s003.docx]

**S3 Table: Additional α-chain TCR Transcripts (CDR3 Region) to those shown in Table2, Expressed in the Aneurysmal Wall of Patient AAA09. These alpha-chain TCR transcripts were unique when compared to each other.**

| **Clone** | **Vα N Jα** | **Transcript Frequency in Specimen** | **p value** | |
| --- | --- | --- | --- | --- |
|  | | | vs.  1/36 | vs.  2/36 |
| **α-chain TCR transcripts amplified by NPA-PCR/Vα-specific PCR** | | |  |  |
| aaa09npa27 | **C A M R E G Y S T L**  tgtgcaatgaga gag ggatacagcaccctc | Vα6.1Jα11  1/36(3%) | ns | ns |
| aaa09npa45 | **C A M R G W G G K L**  tgtgcaatgaga ggatg gggaggaaagctt | Vα6.1Jα23  1/36(3%) | ns | ns |
| aaa09npa35 | **C A M R F F I T Q G G S E K L**  tgtgcaatgaga ttttttata actcagggcggatctgaaaagctg | Vα6.1Jα57  1/36(3%) | ns | ns |
| aaa09npa15 | **C A M R E I I T Q G G S E K L**  tgtgcaatgaga gagattata actcagggcggatctgaaaagctg | Vα6.1Jα57  1/36(3%) | ns | ns |
| aaa09npa30 | **C A A S R G A G N N R K L**  tgtgcagcaagt agggga gctggcaacaaccgtaagctg | Vα8.1Jα38  1/36(3%) | ns | ns |
| aaa09npa12 | **C A L R F R A Y S S A S K I**  tgtgctctg cgattccgtgcc tacagcagtgcttccaagata | Vα9.1Jα3  1/36(3%) | ns | ns |
| aaa09npa42 | **C A Q S P G G Y N K L**  tgtgct cagtccc ctggtggctacaataagctg | Vα9.1Jα4  1/36(3%) | ns | ns |
| aaa09npa32 | **C A L N T G R R A L**  tgtgctcta a acacgggcaggagagcactt | Vα9.1Jα5  1/36(3%) | ns | ns |
| aaa09npa18 | **C A L S L I S S G S A R Q L**  tgtgctcta agtctga tttcttctggttctgcaaggcaactg | Vα9.1Jα22  1/36(3%) | ns | ns |
| aaa09npa34 | **C A L W N A R L**  tgtgctctt tgg aatgccagactc | Vα9.1Jα31  1/36(3%) | ns | ns |
| aaa09npa24 | **C A L S R S G T R L**  tgtgctcta agtcggtccggga ctaggttg | Vα9.1Jα58  1/36(3%) | ns | ns |
| aaa09npa31 | **C A V E D G T G R R A L**  tgtgctgtggag gatggg acgggcaggagagcactt | Vα11.1Jα5  1/36(3%) | ns | ns |
| aaa09npa47 | **C A V D S N S G N T P L**  tgtgctgtg gactc gaattcaggaaacacacctctt | Vα11.1Jα29  1/36(3%) | ns | ns |
| aaa09npa06 | **C A V T S G E S Q G N L**  tgtgctgtt actagcggtga aagccaaggaaatctc | Vα11.1Jα42  1/36(3%) | ns | ns |
| aaa09npa23 | **C A L K T H G Q N F**  tgtgctctt aaaaccc atggtcagaatttt | Vα12.1Jα26  1/36(3%) | ns | ns |
| aaa09npa41 | **C A E S K S S S A S K I**  tgtgcagagagt aaaag cagcagtgcttccaagata | Vα15.1Jα3  1/36(3%) | ns | ns |
| aaa09npa05 | **C A A I G N Q G G K L**  tgtgcagca atagg taaccagggaggaaagctt | Vα21.1Jα23  1/36(3%) | ns | ns |
| aaa09npa20 | **C A A R W A G K S**  tgtgcagca aggtgg gcaggcaaatca | Vα21.1Jα27  1/36(3%) | ns | ns |
| aaa09npa43 | **C V V S A K T G A N N L**  tgtgtggtgagc gcga aaactggggcaaacaacctc | Vα24.1Jα36  1/36(3%) | ns | ns |
| aaa09npa10 | **C A V E G N Q G A Q K L**  tgtgctgtg gaggggaa tcagggagcccagaagctg | Vα28.1Jα55  1/36(3%) | ns | ns |
| aaa09npa19 | **C A G R G L M E Y G N K L**  tgtgcaggg cgaggcctta tggaatatggaaacaaactg | Vα32.1Jα47  1/36(3%) | ns | ns |

|  | | |  | | |  | | | vs.  1/20 | | | vs.  2/20 | | |  |  |  |  |
| --- | --- | --- | --- | --- | --- | --- | --- | --- | --- | --- | --- | --- | --- | --- | --- | --- | --- | --- |
| **α-chain TCR transcripts amplified by single Vα12-specific PCR** | | | | | | | | |  | | |  | | |  |  |  |  |
| aaa09va1220 | | | **C A L G S G G Y Q K V**  tgtgctctg gg ttctgggggttaccagaaagtt | | | Vα12.1Jα13  1/20(5.0%) | | | ns | | | ns | | |  |  |  |  |
| aaa09va1218 | | | **C A L K T H G Q N F**  tgtgctctt aaaaccc atggtcagaatttt | | | Vα12.1Jα26  1/20(5%) | | | ns | | | ns | | |  |  |  |  |
| aaa09va1204 | | | **C A L S G T G T A S K L**  tgtgctctgagc gg taccggcactgccagtaaactc | | | Vα12.1Jα44  1/20(5%) | | | ns | | | ns | | |  |  |  |  |
| aaa09va1209 | | | **C A L Y S G G G A D G L**  tgtgctctc tattcaggaggaggtgctgacggactc | | | Vα12.1Jα45  1/20(5%) | | | ns | | | ns | | |  |  |  |  |
| aaa09va1215 | | | **C A L S E A V P G A N S K L**  tgtgctctgagt gaggccgttc ctggagccaatagtaagctg | | | Vα12.1Jα56  1/20(5%) | | | ns | | | ns | | |  |  |  |  |
|  | | |  | | |  | | | **p value** | | | | | |  |  |  |  |
|  | | |  | | |  | | | vs.  1/21 | | | vs.  2/21 | | |  |  |  |  |
| **α-chain TCR transcripts amplified by single Vα6-specific PCR** | | | | | | | | |  | | |  | | |  |  |  |  |
| aaa09va0618 | | | **C A M P Y G N N R L**  tgtgcaatg cc ctatgggaacaacagactc | | | Vα6.1Jα7  1/21(5%) | | | Ns | | | ns | | |  |  |  |  |
| aaa09va0623 | | | **C A M R E G R G G Y S T L**  tgtgcaatgaga gagggccgggg aggatacagcaccctc | | | Vα6.1Jα11  1/21(5%) | | | Ns | | | ns | | |  |  |  |  |
| aaa09npa45 | | | **C A M R G W G G K L**  tgtgcaatgaga ggatg gggaggaaagctt | | | Vα6.1Jα23  1/21(5%) | | | Ns | | | ns | | |  |  |  |  |
| aaa09va0605 | | | **C A M R R S N Y Q L**  tgtgcaatgaga cgg agcaactatcagtta | | | Vα6.1Jα33  1/21(5%) | | | Ns | | | ns | | |  |  |  |  |
| aaa09va0617 | | | **C A M T S T N Y Q L**  tgtgcaatg acaagcac caactatcagtta | | | Vα6.1Jα33  1/21(5%) | | | Ns | | | ns | | |  |  |  |  |
| aaa09va0624 | | | **C A M R E N S N Y Q L**  tgtgcaatgaga gagaac agcaactatcagtta | | | Vα6.1Jα33  1/21(5%) | | | Ns | | | ns | | |  |  |  |  |
| aaa09va0622 | | | **C A M R E A S S G N T G K L**  tgtgcaatgaga gaggcttc ctctggcaacacaggcaaacta | | | Vα6.1Jα37  1/21(5%) | | | Ns | | | ns | | |  |  |  |  |
| aaa09va0604 | | | **C A M I T N Y G G S Q G N L**  tgtgcaatg attac gaattatggaggaagccaaggaaatctc | | | Vα6.1Jα42  1/21(5%) | | | Ns | | | ns | | |  |  |  |  |
| aaa09va0610 | | | **C A M N P S G G G A D G L**  tgtgcaatg aacccc tcaggaggaggtgctgacggactc | | | Vα6.1Jα45  1/21(5%) | | | Ns | | | ns | | |  |  |  |  |
| aaa09npa15 | | | **C A M R E I I T Q G G S E K L**  tgtgcaatgaga gagattata actcagggcggatctgaaaagctg | | | Vα6.1Jα57  1/21(5%) | | | Ns | | | ns | | |  |  |  |  |
| aaa09npa35 | | | **C A M R F F I T Q G G S E K L**  tgtgcaatgaga ttttttata actcagggcggatctgaaaagctg | | | Vα6.1Jα57  1/21(5%) | | | Ns | | | ns | | |  |  |  |  |
| **α-chain TCR transcripts amplified by single Vα9-specific PCR** | | | | | | | | | vs.  1/19 | | | vs.  2/19 | | |  | | |  |
| aaa09va0915 | | | **C A L R S N D Y K L**  tgtgctcta c gttctaacgactacaagctc | | | Vα9.1Jα20  1/19(5%) | | | ns | | | ns | | |  |  |  |  |
| aaa09va0913 | | | **C A L S L I S S G S A R Q L**  tgtgctctaagt ctga tttcttctggttctgcaaggcaactg | | | Vα9.1Jα22  1/19(5%) | | | ns | | | ns | | |  |  |  |  |
| aaa09va0907 | | | **C A L S T F Y G Q N F**  tgtgctctaagc acctt ctatggtcagaatttt | | | Vv9.1Jα26  1/19(5%) | | | ns | | | ns | | |  |  |  |  |
| aaa09va0916 | | | **C A L S A G N T P L**  tgtgctctaagt g caggaaacacacctctt | | | Vα9.1Jα29  1/19(5%) | | | ns | | | ns | | |  |  |  |  |
| aaa09va0923 | | | **C A L K M N S G N T P L**  tgtgctcta aaaat gaattcaggaaacacacctctt | | | Vα9.1Jα29  1/19(5%) | | | ns | | | ns | | |  |  |  |  |
| aaa09npa34 | | | **C A L W N A R L**  tgtgctctt tgg aatgccagactc | | | Vα9.1Jα31  1/19(5%) | | | ns | | | ns | | |  |  |  |  |
| aaa09va0910 | | | **C A L L T G N Q F**  tgtgctcta ct caccggtaaccagttc | | | Vα9.1Jα49  1/19(5%) | | | ns | | | ns | | |  |  |  |  |
