# Supplementary material for: Clonally expanded alpha-chain T-cell receptor (TCR) transcripts are present in aneurysmal lesions of patients with Abdominal Aortic Aneurysm (AAA)
Source: PLoS One. 2019 Jul 16;14(7):e0218990. doi: 10.1371/journal.pone.0218990 (PMC6634378; doi:10.1371/journal.pone.0218990)
Supplement: S4 Table — These α-chain TCR transcripts were unique when compared to each other. (DOCX) [file pone.0218990.s004.docx]

**S4 Table: Additional α-chain TCR Transcripts (CDR3 Region) to those shown in Table2, Expressed in the Aneurysmal Wall of Patient AAA10. These alpha-chain TCR transcripts were unique when compared to each other.**

| **Clone** | **Vα N Jα** | **Transcript Frequency in Specimen** | **p value** | |
| --- | --- | --- | --- | --- |
|  | | | vs.  1/30 | vs.  2/30 |
| **α-chain TCR transcripts amplified by NPA-PCR/Vα-specific PCR** | | |  |  |
| aaa10npa20 | **C A T A Y Y S G G G A D G L**  tgtgctacg gcctac tattcaggaggaggtgctgacggactc | Vα3.1Jα45  1/30(3%) | ns | ns |
| aaa10npa36 | **C A P G G G F K T**  tgtgct cccggg ggaggcttcaaaact | Vα5.1Jα9  1/30(3%) | ns | ns |
| aaa10npa30 | **C A L A V N N A R L**  tgtgctcta gctgtg aacaatgccagactc | Vα5.1Jα31  1/30(3%) | ns | ns |
| aaa10npa29 | **C A M R E A Y S G N Q F Y**  tgtgcaatgaga gaggcgtact ccggtaaccagttctat | Vα6.1Jα49  1/30(3%) | ns | ns |
| aaa10npa17 | **C A V R S Y N T D K L**  tgtgctgtg aga tcttataacaccgacaagctc | Vα11.1Jα34  1/30(3%) | ns | ns |
| aaa10npa02 | **C A V E S T G G F K T**  tgtgctgtg gagagc actggaggcttcaaaact | Vα13.1Jα9  1/30(3%) | ns | ns |
| aaa10npa31 | **C A V T P T G G G N K L**  tgtgctgtt acccc cacgggaggaggaaacaaactc | Vα13.1Jα10  1/30(3%) | ns | ns |
| aaa10npa39 | **C A G T G Y N F N K F**  tgtgcg gggaccggt tacaacttcaacaaattt | Vα13.1Jα21  1/30(3%) | ns | ns |
| aaa10npa01 | **T D S V F G N V L**  acagactcg gtt tttgggaatgtgctg | Vα13.1Jα35  1/30(3%) | ns | ns |
| aaa10npa24 | **C A A G V Y N Q G G K L**  tgtgcg gccgggg tttataaccagggaggaaagctt | Vα15.1Jα23  1/30(3%) | ns | ns |
| aaa10npa32 | **C A Y R N Y G G S Q**  tgtgcg tatcg gaattatggaggaagccaa | Vα15.1Jα42  1/30(3%) | ns | ns |
| aaa10npa42 | **C A A S P G K N K L**  tgtgcagcaagc ccgggcaa aaacaaactc | Vα17.1Jα10  1/30(3%) | ns | ns |
| aaa10npa12 | **C A T M S G Y G N K L**  tgtgcc accatgtcgggg tatggaaacaagctg | Vα19.1Jα47  1/30(3%) | ns | ns |
| aaa10npa16 | **C A A S G H G P G T Y K Y I F G**  tgtgcagcaagc gggcatggcccaggaacctacaaata catctttgga | Vα21.1Jα34  1/30(3%) | ns | ns |
| aaa10npa41 | **C A A P F G N Q F Y**  tgtgcagcc ccctt cggtaaccagttctat | Vα21.1Jα49  1/30(3%) | ns | ns |
| aaa10npa48 | **C A L W G G G F K T**  tgtgctctc tgggga ggaggcttcaaaact | Vα22.1Jα9  1/30(3%) | ns | ns |
| aaa10npa33 | **C A A E G S S N T G K L**  tgtgcc gcggaaggctcta gcaacacaggcaaacta | Vα27.1Jα37  1/30(3%) | ns | ns |
| aaa10npa10 | **C G T V H T T G T A S K L**  tgcggcaca gtgcacacc accggcactgccagtaaactc | Vα29.1Jα44  1/30(3%) | ns | ns |
|  | | |  | |
| **α-chain TCR transcripts following Vα6-specific PCR** | |  | vs.  1/21 | vs.  2/21 |
| aaa10va0609 | **C A M R E G F S D G Q K L**  tgtgcaatgaga gaggg gttttcagatggccagaagctg | Vα6.1Jα15  1/21(5%) | ns | ns |
| aaa10va0621 | **C A P R E G R N D Y K L**  tgtgca ccgagagagggccgg aacgactacaagctc | Vα6.1Jα20  1/21(5%) | ns | ns |
| aaa10va0619 | **C A M R G N Q G G K L**  tgtgcaatgagg gg taaccagggaggaaagctt | Vα6.1Jα23  1/21(5%) | ns | ns |
| aaa10va0607 | **Y F C P R Q T S G S R L**  tatttctgc ccgcggc aaaccagtggctctaggttg | Vα6.1Jα58  1/21(5%) | ns | ns |
|  |  |  |  |  |
