# Supplementary material for: Clonally expanded alpha-chain T-cell receptor (TCR) transcripts are present in aneurysmal lesions of patients with Abdominal Aortic Aneurysm (AAA)
Source: PLoS One. 2019 Jul 16;14(7):e0218990. doi: 10.1371/journal.pone.0218990 (PMC6634378; doi:10.1371/journal.pone.0218990)
Supplement: S5 Table — These α-chain TCR transcripts were unique when compared to each other. (DOCX) [file pone.0218990.s005.docx]

**S5 Table: Additional α-chain TCR Transcripts (CDR3 Region) to those shown in Table2, Expressed in the Aneurysmal Wall of Patient AAA12. These alpha-chain TCR transcripts were unique when compared to each other.**

| **Clone** | **Vα N Jα** | **Transcript Frequency in Specimen** | **p value** | |
| --- | --- | --- | --- | --- |
|  | | | vs.  1/31 | vs.  2/31 |
| **α-chain TCR transcripts amplified by NPA-PCR/Vα-specific PCR** | | |  |  |
| aaa12npa13 | **C A V D D G G A T N K L**  tgtgctgtg gacgatggt ggtgctacaaacaagctc | Vα11.1Jα32  1/31(3%) | ns | ns |
| aaa12npa44 | **C A V D E G L T G G G N K L**  tgtgctgtg gatgagggg ctcacgggaggaggaaacaaactc | Vα13.1Jα10  1/31(3%) | ns | ns |
| aaa12npa21 | **C A V G G S D S S Y K L**  tgtgctgtg ggcgggtc ggatagcagctataaattg | Vα13.1Jα12  1/31(3%) | ns | ns |
| aaa12npa33 | **C A V E R N D Y K L**  tgtgctgtgga gcgc aacgactacaagctc | Vα13.1Jα20  1/31(3%) | ns | ns |
| aaa12npa06 | **C A V Y N T N A G K S**  tgtgctgt cta taacaccaatgcaggcaaatca | Vα13.1Jα27  1/31(3%) | ns | ns |
| aaa12npa30 | **C A V V G D T G N Q F Y F**  tgtgctgtg gtcgggg acaccggtaaccagttctatttt | Vα13.1Jα49  1/31(3%) | ns | ns |
| aaa12npa35 | **C A Y R E G A Q K L**  tgtgcttat cgcg agggagcccagaagctg | Vα14.1Jα55  1/31(3%) | ns | ns |
| aaa12npa34 | **C A S I R M S S Y K L**  tgtgct tccatta ggatgagcagctataaattg | Vα14.2Jα12  1/31(3%) | ns | ns |
| aaa12npa22 | **C A E S K P G G S Y I P**  tgtgcagagagt aaac caggaggaagctacatacct | Vα15.1Jα6  1/31(3%) | ns | ns |
| aaa12npa15 | **C A L G G A T N K L**  tgtgcc ctcggt ggtgctacaaacaagctc | Vα19.1Jα32  1/31(3%) | ns | ns |
| aaa12npa10 | **C A V S S P D T D K L**  tgtgctgtc agttcacccg acaccgacaagctc | Vα19.1Jα34  1/31(3%) | ns | ns |
| aaa12npa46 | **C A A R D D N Q G G K L**  tgtgcagca agggatg ataaccagggaggaaagctt | Vα21.1Jα23  1/31(3%) | ns | ns |
| aaa12npa42 | **C A A R G S G G S N Y K L**  tgtgcagca agaggg agtggaggtagcaactataaactg | Vα21.1Jα54  1/31(3%) | ns | ns |
| aaa12npa17 | **C V V S T G G G N K L**  tgtgtggtgagc acgggaggaggaaacaaactc | Vα24.1Jα10  1/31(3%) | ns | ns |
| aaa12npa37 | **C V V I S R M D S S Y K L**  tgtgtggtg atctcca ggatggatagcagctataaattg | Vα24.1Jα12  1/31(3%) | ns | ns |
| aaa12npa43 | **C V V T G S S N T G K L**  tgtgtggtg accggctcta gcaacacaggcaaacta | Vα24.1Jα37  1/31(3%) | ns | ns |
| aaa12npa08 | **C V V S A G T Y K Y**  tgtgtggtgagc g caggaacctacaaatac | Vα24.1Jα40  1/31(3%) | ns | ns |
| aaa12npa05 | **C G A D P I F S N S G Y A L**  tgtggagcagac cccattttt tcaaattccgggtatgcactc | Vα26.1Jα41  1/31(3%) | ns | ns |
| aaa12npa03 | **C R L R G G G A D G L**  tgt agactcaggggg ggaggtgctgacggactc | Vα26.1Jα45  1/31(3%) | ns | ns |
| aaa12npa41 | **C G T E G A N D Y K L**  tgcggcacagag ggtg ctaacgactacaagctc | Vα29.1Jα20  1/31(3%) | ns | ns |
| aaa12npa45 | **C G T E G V N D Y K L**  tgcggcacagag ggggta aacgactacaagctc | Vα29.1Jα20  1/31(3%) | ns | ns |
| aaa12npa01 | **C A G Q G A Q K L**  tgtgcaggg cagggagcccagaagctg | Vα32.1Jα55  1/31(3%) | ns | ns |
|  |  |  |  | |
