# Supplementary material for: Clonally expanded alpha-chain T-cell receptor (TCR) transcripts are present in aneurysmal lesions of patients with Abdominal Aortic Aneurysm (AAA)
Source: PLoS One. 2019 Jul 16;14(7):e0218990. doi: 10.1371/journal.pone.0218990 (PMC6634378; doi:10.1371/journal.pone.0218990)
Supplement: S6 Table — These α-chain TCR transcripts were unique when compated to each other. (DOCX) [file pone.0218990.s006.docx]

**S6 Table. α-chain TCR Transcripts (CDR3 Region) Identified in PBMC from Normal Donors**

| **Clone** | **Vα N Jα** | **Transcript Frequency in Specimen** | **p value** | |
| --- | --- | --- | --- | --- |
|  | | | vs.  1/28 | vs.  2/28 |
| **α-chain TCR transcripts from Normal Donor 1 amplified by NPA-PCR/Vα-specific PCR is shown in Table 3 of the paper.** | | |  |  |
|  |  |  | vs. 1/47 | vs. 2/47 |
| **α-chain TCR transcripts from Normal Donor 2 amplified by NPA-PCR/Vα-specific PCR** | | |  |  |
| ND6LA01 | **C A L D R E G A G S Y Q L T F** tgtgctctagac cgggag ggggctgggagttaccaactcactttc | Vα6.3Jα28.1  1/47 | ns | ns |
| ND6LA02 | **C G A D A D I L N A G G T S Y G K L T F**  tgtggagcaga gcagatatact taatgctggtggtactagctatggaaagctgacattt | Vα34.1Jα52.1  1/47 | ns | ns |
| ND6LA03 | **C A A S E N G G A T N K L I F** tgtgcagcaag cgaga atggcggtgctacaaacaagctcatcttt | Vα23.1Jα32.1  1/47 | ns | ns |
| ND6LA04 | **C G P F P G G G A D G L T F**  tgtgga ccttttc caggaggaggtgctgacggactcaccttt | Vα34.1Jα45.1  1/47 | ns | ns |
| ND6LA06 | **C A V D A E S G A G S Y Q L T F** tgtgccgtggac gcagag tctggggctgggagttaccaactcactttc | Vα39.1Jα28.1  1/47 | ns | ns |
| ND6LA09 | **C L V G V D S S G S A R Q L T F** tgcctcgtgggtg tcga ttcttctggttctgcaaggcaactgaccttt | Vα4.1Jα22.1  1/47 | ns | ns |
| ND6LA10 | **C A V P R T G A N S K L T F** tgtgctgt accccg tactggagccaatagtaagctgacattt | Vα22.1Jα56.1  1/47 | ns | ns |
| ND6LA11 | **C A M I P R G G S Y I P T F** tgtgcaatga tcccccg aggaggaagctacatacctacattt | Vα14.1Jα6.1  1/47 | ns | ns |
| ND6LA13 | **C A V E G T G G F K T I F** tgtgctgtg gaggg tactggaggcttcaaaactatcttt | Vα22.1Jα9.1  1/47 | ns | ns |
| ND6LA14 | **C A A M E Y G N K L V F** tgtgctg cca tggaatatggaaacaaactggtcttt | Vα22.1Jα47.1  1/47 | ns | ns |
| ND6LA16 | **C G A D N A G G T S Y G K L T F** tgtgga gcagacaac gctggtggtactagctatggaaagctgacattt | Vα34.1Jα52.1  1/47 | ns | ns |
| ND6LA18 | **C L L G K E T S G S R L T F** tgtcttctggg ga aagaaaccagtggctctaggttgaccttt | Vα40.1Jα58.1  1/47 | ns | ns |
| ND6LA20 | **C G T A Y S G A G S Y Q L T F** tgcggc acag catactctggggctgggagttaccaactcactttc | Vα30.1Jα28.1  1/47 | ns | ns |
| ND6LA22 | **C A V R N S G N T P L V F** tgtgct gtgc ggaattcaggaaacacacctcttgtcttt | Vα20.1Jα29.1  1/47 | ns | ns |
| ND6LA23 | **C A V V P N Y G G A T N K L I F** tgtgctgtg gttcc gaattatggcggtgctacaaacaagctcatcttt | Vα22.1Jα32.1  1/47 | ns | ns |
| ND6LA24 | **C A A A I S G N T G K L I F** tgtgcagca gcaat ctctggcaacacaggcaaactaatcttt | Vα23.1Jα37.1  1/47 | ns | ns |
| ND6LA25 | **C G T S D P N S N S G Y A L N F**  tgcggc acctccgatcc gaactcaaattccgggtatgcactcaacttc | Vα30.1Jα41.1  1/47 | ns | ns |
| ND6LA26 | **C A L S G R N N N A R L M F**  tgtgctct gagtggccg gaataacaatgccagactcatgttt | Vα19.1Jα31.1  1/47 | ns | ns |
| ND6LA27 | **R V V S A Q N N N A R L M F**  **c** gtgtggtgagcgcgcaa aataacaatgccagactcatgttt | Vα10.1Jα31.1  1/47 | ns | ns |
| ND6LA28 | **C V V I F A S G G S Y I P T F** tgtgtg gtgatctt tgcatcaggaggaagctacatacctacattt | Vα10.1Jα6.1  1/47 | ns | ns |
| ND6LA29 | **C A G R N S N Y Q L I W**  tgtgct gggcgga atagcaactatcagttaatctgg | Vα35.1Jα33.1  1/47 | ns | ns |
| ND6LA30 | **C A E N S G G S Y I P T F**  tgtgctg agaac tcaggaggaagctacatacctacattt | Vα35.1Jα33.1  1/47 | ns | ns |
| ND6LA31 | **C A M R G F Q Y N T D K L I F** tgtgcaatgagag ggtttcaa tataacaccgacaagctcatcttt | Vα14.3Jα34.1  1/47 | ns | ns |
| ND6LA33 | **C A A S N H G N K L V F** tgtgcagcaagca atc atggaaacaaactggtcttt | Vα23.1Jα47.1  2/47 | ns | ns |
| ND6LA35 | **C G P F P G G G A D G L T F**  tgtgga ccttttc caggaggaggtgctgacggactcaccttt | Vα34.1Jα45.1  1/47 | ns | ns |
| ND6LA36 | **C A V G T N N A G N M L T F** tgtgctgtg gggacc aataatgcaggcaacatgctcaccttt | Vα22.1Jα39.1  1/47 | ns | ns |
| ND6LA38 | **C A V N N A R L M F**  tgtgctgtg aacaatgccagactcatgttt | Vα22.1Jα31.1  1/47 | ns | ns |
| ND6LA40 | **C A P G T S G A G S Y Q L T F** tgtgct cccgggact tctggggctgggagttaccaactcactttc | Vα6.3Jα28.1  1/47 | ns | ns |
| ND6LA41 | **C A A S R P P N F G N E K L T F** tgtgcagcaag ccgccccc ctaactttggaaatgagaaattaaccttt | Vα23.1Jα48.1  1/47 | ns | ns |
| ND6LA43 | **C A V L R S Y N T D K L I F**  tgtgcc gtgttgcga tcttataacaccgacaagctcatcttt | Vα39.1Jα34.1  2/47 | ns | ns |
| ND6LA46 | **C V V S F D T G G F K T I F** tgtgtg gtgagctttg atactggaggcttcaaaactatcttt | Vα10.1Jα9.1  1/47 | ns | ns |
| ND6LA47 | **C A V E M T T D S W G K L Q F** tgtgctgtg gaga tgacaactgacagctgggggaaattgcagttt | Vα22.1Jα24.1  1/47 | ns | ns |
| ND6LA48 | **C A V N D Y K L S F**  tgtgctgt c aacgactacaagctcagcttt | Vα22.1Jα20.1  1/47 | ns | ns |
| ND6LA49 | **C A V S N D Y K L S F**  tgtgctgt c tctaacgactacaagctcagcttt | Vα22.1Jα20.1  1/47 | ns | ns |
| ND6LA50 | **C G A D N T G G F K T I F** tgtgga gcagacaac actggaggcttcaaaactatcttt | Vα34.1Jα9.1  1/47 | ns | ns |
| ND6LA51 | **C A V S R S G A N S K L T F** tgtgccgtga gtcgatca ggagccaatagtaagctgacattt | Vα12-2.01Jα56.1  1/47 | ns | ns |
| ND6LA53 | **C G L M G G G S E K L V F** tgtgga cttatggg gggcggatctgaaaagctggtcttt | Vα34.1Jα57.1  1/47 | ns | ns |
| ND6LA54 | **C G A D K G N T D K L I F** tgtgga gcagacaaaggg aacaccgacaagctcatcttt | Vα34.1Jα34.1  1/47 | ns | ns |
| ND6LA56 | **C V V S W N N N A R L M F** tgtgtg gtgagctg gaataacaatgccagactcatgttt | Vα10.1Jα31.1  1/47 | ns | ns |
| ND6LA57 | **C A P G E T S G S R L T F** tgtgct ccgggg gaaaccagtggctctaggttgaccttt | Vα22.1Jα58.1  1/47 | ns | ns |
| ND6LA59 | **C A L D R Y S S A S K I I F** tgtgctctagac c ggtacagcagtgcttccaagataatcttt | Vα6.3Jα3.1  1/47 | ns | ns |
| ND6LA60 | **C A V Q A Y G G S Q G N L I F** tgtgctgtgcagg cc tatggaggaagccaaggaaatctcatcttt | Vα20.1Jα42.1  1/47 | ns | ns |
| ND6LA61 | **C A E S M A N Q G G K L I F** tgtgcagaga gtatggc taaccagggaggaaagcttatcttc | Vα5.1Jα23.1  1/47 | ns | ns |
| NDL6LA64 | **C A V E R F T D S W G K L Q F** tgtgctgtg gagaggttc actgacagctgggggaaattgcagttt | Vα22.1Jα24.2  1/47 | ns | ns |
| ND6LA65 | **C G A V P N D Y K L S F** tgtggagc ggttc ctaacgactacaagctcagcttt | Vα34.1Jα20.1  1/47 | ns | ns |
|  |  |  |  |  |
|  |  |  | vs.  1/50 | vs.  2/50 |
| **α-chain TCR transcripts from Normal Donor 3 amplified by NPA-PCR/Vα-specific PCR** | | |  |  |
| NDA11.1 | **Y L C G A D R A G G S E K L V F** tacctctgtgga gcagacagagc gggcggatctgaaaagctggtcttt | Vα34.1Jα57.1  1/50 | ns | ns |
| NDA11.2 | **C A E S K E H L L S** tgtgcagaga gtaagga acacctcttgtct | Vα5.1Jα29.1 1/50 | ns | ns |
| NDA11.3 | **Y F C A L R T G N Q F Y F** tacttctgtgct ctgagg accggtaaccagttctatttt | Vα19.1Jα49.1 1/50 | ns | ns |
| NDA11.4 | **F C A A S S D Y K L S F** tctgtgcagcaa gtag cgactacaagctcagcttt | Vα13-1.1Jα20.1  1/50 | ns | ns |
| NDA11.5 | **Y F C A L T S G T Y K Y I F** tacttctgtgct cta acctcaggaacctacaaatacatcttt | Vα35.1Jα40.1  1/50 | ns | ns |
| NDA11.6 | **C A Y R S Q H A G N N R K L I W**  tgtgctt ataggagtcaacac ctggcaacaaccgtaagctgatttgg | Vα38-2/Jα38.1 1/50 | ns | ns |
| NDA11.7 | **C A L S G D N D M R F** tgtgctct gagtgggg acaatgacatgcgcttt | Vα9-2.1Jα43.1  1/50 | ns | ns |
| NDA11.8 | **C A E S P N Y G G S Q G N L V F** tgtgcagaga gtccga attatggaggaagccaaggaaatctcgtcttt | Vα5.1Jα42.1 1/50 | ns | ns |
| NDA11.9 | **C A E N P D A G G T S Y G K L T F** tgtgcagaga atccggat ctggtggtactagctatggaaagctgacattt | Vα13-2.1Jα53.1 1/50 | ns | ns |
| NDA11.10 | **C A E S I R D Y K L S F**  tgtgcagaga gtatacgg gactacaagctcagcttt | Vα5.1Jα20.1 1/50 | ns | ns |
| NDA11.11 | **C A A S D Y G Q N F V F** tgtgcagcaagcg actatggtcagaattttgtcttt | Vα29Jα26.1 1/50 | ns | ns |
| NDA11.12 | **C A A S A Y P G S A R Q L T F** tgtgcagcaagcg cg tacc ctggttctgcaaggcaactgaccttt | Vα29Jα22.1  1/50 | ns | ns |
| NDA11.13 | **C A P Q Y S G A G S Y Q L T F** tgtgct cccca atactctggggctgggagttaccaactcactttc | Vα36/DV7.1Jα28.1 2/50 | ns | ns |
| NDA11.15 | **C G A D L G G N T P L V F**  tgtgga gcagacttagg aggaaacacacctcttgtcttt | Vα34.1Jα29.1 2/50 | ns | ns |
| NDA11.17 | **C A A R R R N A G N M L T F** gtgcagca ggaagaaga aatgcaggcaacatgctcaccttt | Vα29Jα39.1 1/50 | ns | ns |
| NDA11.18 | **C A V T V Y N F N K F Y F**  tgtgctgtg accgtat acaacttcaacaaattttacttt | Vα8-4.1Jα21.1 1/50 | ns | ns |
| NDA11.19 | **C A V R D R Q F A G T A L I**  tgcgctgtgaga ga tagg caggcaggaactgctctgatcttt | Vα1-1.1Jα15.1 2/50 | ns | ns |
| NDA11.21 | **C V V S A K C Q T H V**  tgtgtggtgagcg c ca aatgccagactcatgttt | Vα10.1Jα31.1 2/50 | ns | ns |
| NDA11.23 | **C A G L I G N E K L T F**  tgtgcaggg ttaatc ggaaatgagaaattaaccttt | Vα25.1Jα48.1 1/50 | ns | ns |
| NDA11.24 | **C A T D G W G S Q G N L I F**  tgtgctac ggacgggtgg ggaagccaaggaaatctcatcttt | Vα17.1Jα42.1 2/50 | ns | ns |
| NDA11.25 | **C V V S A G W A G G Y Q K V A F** tgtgtggtgagcg cgggatggg ctgggggttaccagaaagttgccttt | Vα10.1Jα13.1 1/50 | ns | ns |
| NDA11.26 | **C V V S A L T G G G N K L T F**  tgtgtggtgagcg cg ctcacgggaggaggaaacaaactcaccttt | Vα10.1Jα10.1 1/50 | ns | ns |
| NDA11.27 | **C G T E T W T G A G S Y Q L T F**  tgcggc acagagacctgga ctggggctgggagttaccaactcactttc | Vα30.1Jα28.1 1/50 | ns | ns |
| NDA11.28 | **C V V S G G I I R R K L H T Y I**  tgtgtg gtgagcggggggat catcaggaggaagctacatacctacatt | Vα10.1Jα6.1 1/50 | ns | ns |
| NDA11.29 | **C G A D R K A A E T S**  tgtgga gcagaccgg aaagcagcggagacaagc | Vα34.1Jα46.1 1/50 | ns | ns |
| NDA11.30 | **C A L Q G G S Q G N L I F**  tgtgct ctacaa ggaggaagccaaggaaatctcatcttt | Vα6.1Jα42.1 1/50 | ns | ns |
| NDA11.31 | **C G T G G G G T S Y G K L T F**  tgcggc acaggagg tggtggtactagctatggaaagctgacattt | Vα30.1Jα53.1 1/50 | ns | ns |
| NDA11.32 | **C A G R N N N A R L M F**  tgtgct gggcg gaataacaatgccagactcatgttt | Vα36Jα31.1 1/50 | ns | ns |
| NDA11.33 | **C A L R T A V L P R**  tgtgct cttc gtacagcagtgcttccaaga | Vα6.1Jα3.1 1/50 | ns | ns |
| NDA11.34 | **C A A S D L F S G Y S T L T F**  tgtgcagcaagcg atctatt ttcaggatacagcaccctcaccttt | Vα29Jα11.1 1/50 | ns | ns |
| NDA11.35 | **C G T V S S G T A S K L T F**  tgcggc acagtttcct ccggcactgccagtaaactcaccttt | Vα30.1Jα44.1 2/50 | ns | ns |
| NDA11.36 | **C A R E E Q C F Q D N L**  tgtgct cgggagg agcagtgcttccaagataatctt | Vα6.1Jα3.1 1/50 | ns | ns |
| NDA11.38 | **C A T N Q A G T A L I F**  tgtgctac gaac caggcaggaactgctctgatcttt | Vα17.1Jα15.1 1/50 | ns | ns |
| NDA11.39 | **C G A D L D R I L S**  tgtgga gcagacttaga cagaattttgtcttt | Vα34.1Jα26.1 1/50 | ns | ns |
| NDA11.40 | **C A G N N N D M R F**  tgtgct ggg aataacaatgacatgcgcttt | Vα35.1Jα43.1 1/50 | ns | ns |
| NDA11.41 | **C A T E D S N Y Q L I W**  tgtgctac gga ggatagcaactatcagttaatctgg | Vα17.1Jα33.1 1/50 | ns | ns |
| NDA11.42 | **C G T V T T G T A S K L T F**  tgcggc acagtgac taccggcactgccagtaaactcaccttt | Vα30.1Jα44.1 1/50 | ns | ns |
| NDA11.44 | **C A L A S M D S S Y K L I F**  tgtgct ctagcttca atggatagcagctataaattgatcttc | Vα6.1Jα12.1 1/50 | ns | ns |
| NDA11.46 | **C A L R L S E T C I**  tgtgct ct aag gctttcagaaacttgtattt | Vα16.1Jα8.1 1/50 | ns | ns |
| NDA11.50 | **C A T A N N F N K F Y F**  tgtgctac ggcca acaacttcaacaaattttacttt | Vα17.1Jα21.1 1/50 | ns | ns |
| NDA11.51 | **C A V E P P T G R R A L T F**  tgtgctgtg gagccccca acgggcaggagagcacttactttt | Vα22.1Jα5.1 1/50 | ns | ns |
| NDA11.52 | **C G A V F L G A Q K L V F**  tgtgga gcagtctttct gggagcccagaagctggtattt | Vα34.1Jα55.1 1/50 | ns | ns |
| NDA11.53 | **C A A S P R G S Q G N L I F**  tgtgcagcaagc ccgag aggaagccaaggaaatctcatcttt | Vα29Jα42.1 1/50 | ns | ns |
| NDA11.54 | **Y F C A G T Y K Y I F**  tacttctgtgct g gaacctacaaatacatcttt | Vα19.1Jα40.1 1/50 | ns | ns |
